# Recent Progress in Small Spirocyclic, Xanthene-Based Fluorescent Probes

**DOI:** 10.3390/molecules25245964

**Published:** 2020-12-16

**Authors:** Sascha G. Keller, Mako Kamiya, Yasuteru Urano

**Affiliations:** 1 Graduate School of Medicine, The University of Tokyo, 7-3-1 Hongo, Bunkyo-ku, Tokyo 113-0033, Japan; skeller@m.u-tokyo.ac.jp (S.G.K.); mkamiya@m.u-tokyo.ac.jp (M.K.); 2 Graduate School of Pharmaceutical Sciences, The University of Tokyo, 7-3-1 Hongo, Bunkyo-ku, Tokyo 113-0033, Japan; 3 AMED-CREST, Japan Agency for Medical Research and Development, 1-7-1 Otemachi, Chiyoda-ku, Tokyo 100-0004, Japan

**Keywords:** fluorescence, xanthene-based, spirocyclic, fluorescein, rhodol, rhodamine

## Abstract

The use of fluorescent probes in a multitude of applications is still an expanding field. This review covers the recent progress made in small molecular, spirocyclic xanthene-based probes containing different heteroatoms (e.g., oxygen, silicon, carbon) in position 10′. After a short introduction, we will focus on applications like the interaction of probes with enzymes and targeted labeling of organelles and proteins, detection of small molecules, as well as their use in therapeutics or diagnostics and super-resolution microscopy. Furthermore, the last part will summarize recent advances in the synthesis and understanding of their structure–behavior relationship including novel computational approaches.

## 1. Introduction

**Scheme 1:**
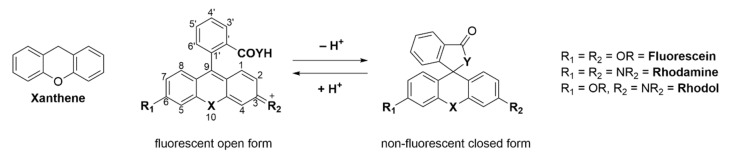
Xanthene, the core-structure of fluorescein and the equilibrium of the open and the closed form of a spirocyclic dye. X = O, S, Si(CH_3_)_2_, C(CH_3_)_2_ and others; Y = O, NH.

Small, spirocyclic, xanthene-based fluorescence probes have become powerful tools for a large variety of bioimaging applications. Adolf von Bayer first synthesized fluorescein in 1871 from phthalic anhydride and resorcinol in the presence of zinc chloride [1]. Since then, fluorescein and its plethora of congeners manifested in a new and versatile branch of research [2]. Nowadays there are many different classes of fluorophores, ranging from large fluorescent proteins (i.e., green fluorescent protein (GFP), yellow fluorescent protein (YFP)) [3,4,5] over small fluorescent molecules (i.e., coumarins [6,7], naphtalimides [8,9,10], BODIPYs [11,12,13,14], cyanines [15,16] or fluoresceins/rhodamines [17,18,19,20]) to fluorescent quantum dots [21,22]. Three main classes exist: fluorescein (contains two phenol groups), rhodamine (contains two aniline groups), and rhodol (contains one phenol group and one aniline group) [23]. Commercialized fluorescein/rhodamine-based dyes (e.g., MitoTracker [24]) are being used daily in a vast amount of chemical, biological or medical research laboratories. These molecules are utilized to visualize and quantify a multitude of static or dynamic processes in living cells due to their high sensitivity, real-time detection, and non-destructive fast analysis [25,26,27]. The probes can target a variety of biological targets due to their structural adjustability [28,29,30,31,32]. Fluoresceins/rhodamines are specifically interesting because of their ability to form a non-fluorescent spirocyclic and a fluorescent open form which can be controlled by external triggers like pH, chelation, or an enzymatic reaction [33]. Furthermore, the ease of chemical modification, good water solubility, high stability and brightness makes them the most popular dyes used in fluorescence imaging. The main chromophore of fluorescein/rhodamine is its xanthene-core, which contains a bridging oxygen-atom in position 10′ (Scheme 1) [34,35]. This bridging atom rigidifies the structure to maximize the π-conjugation within the molecule and thus its fluorescence [19,36,37,38]. However, the absorption and emission of fluoresceins/rhodamines lie within the range of 500–600 nm, which limits their use in multicolor or in vivo imaging. The change of the bridging O-atom to other atoms (C, Si, P, Se, S, Ge, or N) shifts the molecules absorbance maxima to the near-infrared region (NIR) [39]. Many dyes have been developed that cover different parts of the spectrum [40].

Spirocyclic, xanthene-based probes usually have a hydroxy- or carboxy-moiety or other intramolecular nucleophiles at the 2′ position of a pendant aromatic ring at the C-9′-atom of the xanthene core (Scheme 1) [41,42]. This intramolecular nucleophilic-moiety attacks the sp_2_-hybridized C-9′-atom and forms the colorless spirocyclic form. We previously determined the pH-value at which 50% of the molecules are present in the non-fluorescent form as the pK_cycl_ value [43]. Chemical modification of the xanthene core, intramolecular nucleophiles, and the pendant aromatic ring can significantly alter that value [44,45]. This pK_cycl_ value is especially important if the dye should be highly fluorescent at a certain pH, such as the physiological pH of 7.4 for example. On the other hand, for high resolution microscopy it is important that the pK_cycl_ value is much lower, since only a tiny fraction should be fluorescent at a given time so single molecules can be detected [46,47]. 

Another advantage of these dyes is that they can be caged by reacting their aniline or phenol groups with, for example, moieties which are reactive towards biologically relevant molecules or proteins via ether, amide, or ester bonds. This caging alters the fluorescence behavior of the fluorophore turning it non-fluorescent and only after reaction with the desired target is its bright fluorescence restored [48,49,50]. This target-oriented fluorescence activation is what makes these dyes so interesting, as it often results in high signal-to-noise ratios because of little to no nonspecific activation. Targets can range from enzymes to small molecules, organelles, metal ions or simply the pH of the surrounding environment. Moreover, structural changes in the probe itself alter its spectroscopic properties, which allows for fine tuning of the excitation and emission wavelengths [51,52]. For example, many fluorescein/rhodamine analogues have been developed that contain a different heteroatom (e.g., Si, C, P, Se) than oxygen in the 10′-position, resulting in large shifts of their emission and excitation maxima and hence different colors [39,53]. These probes can then be used simultaneously, and different targets can be targeted at the same time. Furthermore, a shift to the near infrared (NIR) region allows deeper penetration of tissue, as well as less photodamage, given the lower energy of the light [54,55].

Several different signal-conversion mechanisms have been utilized. These include Förster resonance energy transfer (FRET) [53], intramolecular charge transfer (ICT) [56], photo-induced electron transfer (PeT) [57,58] and restriction of intramolecular motion (RIM) [59]. 

Among these signal-conversion mechanisms, this review will focus on intramolecular spirocyclization, and introduce recent progress in the use and development of spirocyclic, xanthene-based fluorescent probes within the last three years. First the interactions of probes with enzymes and targeted labeling of organelles and proteins will be covered. Then we will discuss novel methods for the detection of small molecules as well as their use in therapeutics or diagnostics and super-resolution microscopy. The final section will summarize recent advances in the synthesis and understanding of their structure including novel computational approaches. Even though a lot of progress has been achieved, pH-sensing [60,61,62,63,64], metal ion detection [65,66,67,68,69,70,71,72], and the use of nanoparticles [21,73,74,75,76,77,78] or any other solid support [79,80,81,82] will be omitted, given the scope of this review.

## 2. Applications

### 2.1. Enzyme Activation

The detection of enzymatic activity in living cells has become an important field of modern research [83]. Enzymes hold important roles in many physiological, pathological, and pharmacological processes. It is widely accepted that some enzymes are causally related to a variety of cancers. For example, γ-glutamyl-transpeptidase is expressed in high levels in several cancers, including liver, cervical and ovarian cancers [84,85,86,87]; enhanced enzymatic activities of alkaline phosphatase can be detected in some bone cancers [88]; β-galactosidase shows increased activity in primary ovarian cancers [89]. Localizing and determining the expression levels of these enzymes in live cancer cells is of great importance for diagnosing cancer in its early stages and for monitoring the efficacy of therapies. Enzymes specifically catalyze the conversion of certain substrates to their products. A probe bound to one of these substrates will remain non-fluorescent, but upon reaction with the desired enzyme, it will restore its fluorescence. These “turn-on”-probes can achieve high signal-to-noise ratios. In some cases, a linker between the substrate and the fluorophore is desired, to reduce steric hindrance with bulky fluorophores that could hinder the binding of the conjugated substrate into the active pocket of the enzyme [90]. Another approach is the use of non-substrate-based probes, which are bound to the inhibitors of enzymes or their natural ligands. The difficulty here lies in the fact that it is challenging to discriminate between bound and non-bound probes. Probes have been developed that turn fluorescent after binding to the protein of interest [91]. Since the molecule is bound to the enzyme, enzyme activation in real-time can be monitored and its activation and localization can be studied dynamically. 

In this context Johnsson et al. developed a novel Si-based probe for the live-cell imaging of BACE1, an aspartic protease, which is involved in the development of Alzheimer’s disease due to a deregulated Amyloid β (Aβ) homeostasis [92]. 

**Figure 1:**
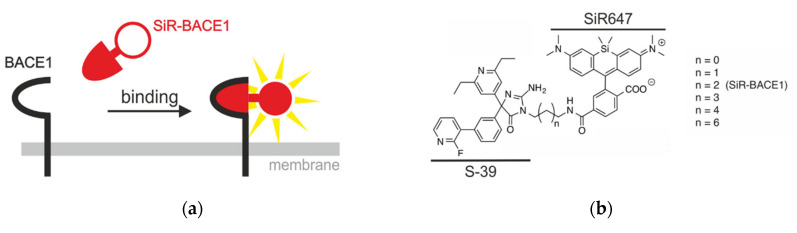

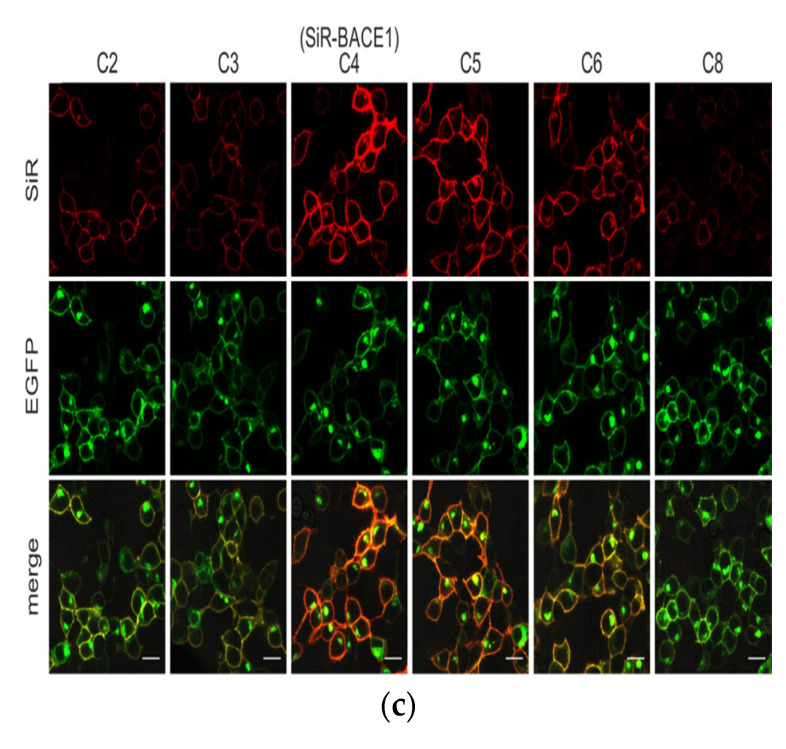
Si-based probe for the imaging of BACE1: (**a**) The activation mechanism of the probe; (**b**) the structure of the probe **SiR-BACE1** and its analogues; (**c**) BACE1-EGFP transiently transfected HEK293T cells, stained with conjugates with different linker lengths (C2–C6, C8). Adapted with permission from [92]. Copyright (2018) American Chemical Society.

A two-step proteolysis from amyloid precursor protein (APP) generates Aβ [93,94,95]. The preclinical BACE1 inhibitor S-39 was fused to the Si-rhodamine SiR647 via diamine linkers with different lengths (2–8 CH_2_ groups, Figure 1b). Their best probe, SiR-BACE1 with a (CH_2_)_4_-linker, showed excellent selectivity, high fluorescence and low nanomolar inhibitory properties (Figure 1c). This linker allowed the fluorophore to be brought close enough to the protein surface to promote fluorescence to be turned on (Figure 1a). Live cell imaging was performed and the surface mobility and diffusion patterns of BACE1 were studied for the first time.

**Figure 2:**
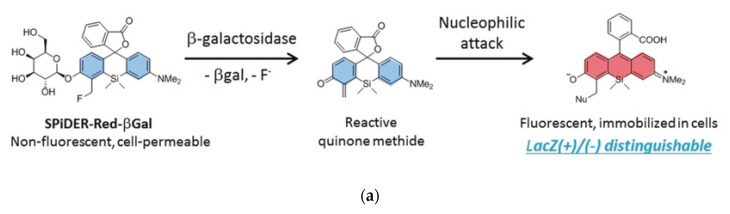

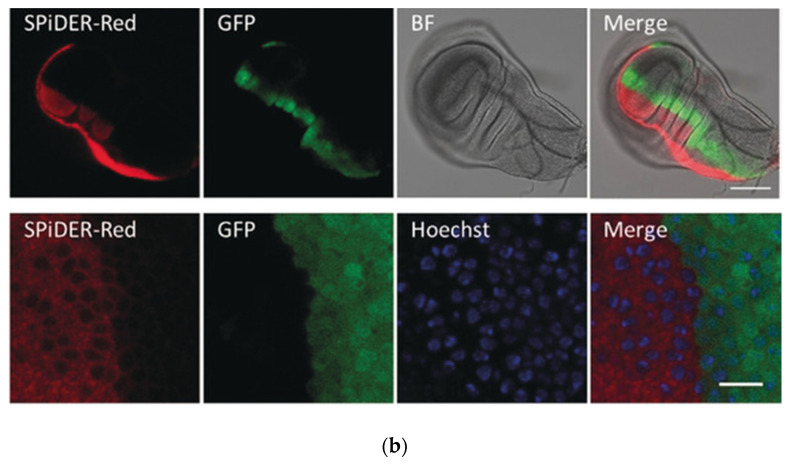
**SPiDER-Red-βGal** for the sensing of LacZ(+) cells: (**a**) Enzymatic activation of **SPiDER-Red-βGal** by β-galactosidase; (**b**) Fluorescence imaging of live *en-lacZ/dpp*-GFP Drosophila third larval wing discs after incubation with **SPiDER-Red-βGal** (top) and additional Hoechst 33342 (bottom). Adapted with permission from [102]. Scale bars = 10 µm.

The β-galactosidase encoding lacZ gene of Escherichia coli has been established in bacteria and animals as a powerful reporter [96]. Even though there are several fluorophores available to detect β-galactosidase, these probes are not suitable to label lacZ-positive cells with single-cell resolution in living organisms [97,98,99,100]. Our group has recently developed the new class of functionalized fluorogenic substrates for β-galactosidase, SPiDER-βGal, which can detect β-galactosidase and label the expressing cell via quinone methide chemistry [101]. Since SPiDER-βGal emits green fluorescence at around 550 nm and green fluorescent protein (GFP), also widely used in fluorescence imaging, emits at around 510 nm, a combination of both is difficult. Hence, probes with a more red-shifted fluorescence are desired. We developed the red-emitting SPiDER-Red-βGal, which contains a 2′COOH-4CH_2_F-Sirhodol as the core fluorophore and a 2′-carboxy group as an intramolecular nucleophile to adapt the spirocyclic non-fluorescent form at physiological pH (Figure 2a) [102]. 

Upon cleavage of the βGal-moiety the reactive quinone methide can react with nucleophilic functional groups within the cell (e.g., cysteines or lysins of proteins) and hence be immobilized. Indeed, the probe was successfully used to observe lacZ-positive cells in Drosophila wing discs together with GFP (Figure 2b). Mice expressing lacZ and GFP and pyramidal neurons were also successfully evaluated. Although GFP stained neurons more clearly, future optimization might improve the image quality and importantly, unspecific staining was not observed.

**Figure 3:**
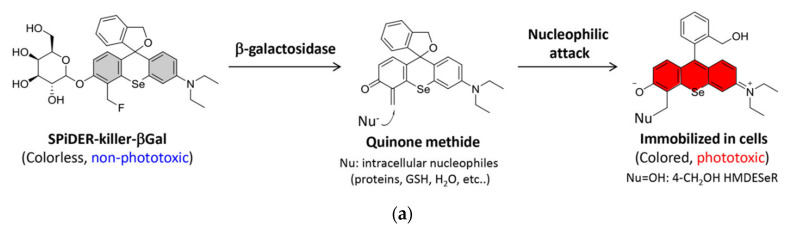

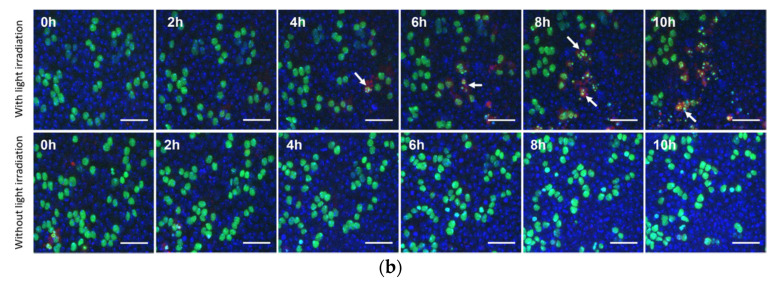
The novel photosensitizer **SPiDER-killer-βGal**: (**a**) Photosensitizer activatable by β-galactosidase (**SPiDER-killer-βGal**), and its reaction scheme with the enzyme; (**b**) Time-lapse in vivo fluorescence imaging of flip-out clones of live pupal notum with (top) and without (bottom) irradiation. Adapted with permission from [103]. Further permissions related to the material excerpted should be directed to the ACS. Scale bars = 30 µm.

Related to the SPiDER-red probe a novel activatable selenorhodol-containing photosensitizer probe SPiDER-killer-βGal was developed by our group (Figure 3a) [103]. Upon irradiation, free radicals or singlet oxygen (^1^O_2_) are produced, leading to cell death, similar to genetically encodable photosensitizer proteins, such as KillerRed or MiniSOG [104,105,106]. The new probe also targets lacZ positive cells but in contrast to SPiDER-Red-βGal the new probe can be used for their ablation. This process in general describes the removal of tissue and may be achieved by different means. Although similar photosensitizers that specifically react with lacZ(+) cells have been reported, their phototoxic form tends not to stay within the cell. This results in a leakage of phototoxic products from the target cells during prolonged incubation. The newly developed probe, however, selectively labels lacZ-expressing cells, with the same methide chemistry as SPiDER-Red-βGal. The phototoxic products are only be produced inside the cell and allow selective killing of β-galactosidase expressing cells with single-cell resolution in vivo, as was demonstrated in a Drosophila pupal notum (Figure 3b). Only the nota subjected to light irradiation (Xe lamp 550 nm) showed a considerable number of apoptotic cells, which were stained with VC3Ai, a red marker for apoptotic cells.

Next, we established a molecular design strategy to obtain activatable probes for carboxypeptidases (CP) [107]. These Enzymes are a family of proteases that cleave one or more amino acids from the C-terminal of peptides or proteins [108]. Furthermore, expression levels of CPs are varied in several diseases and cancers [109,110,111,112]. Detection methods for their activities are limited and fluorescent probes are rare, partly because of the difficulty in designing suitable substrate analogues. Our group managed to develop fluorescent probes for two clinically important CPs (carboxypeptidase A (CPA: recognizes C-terminal bulky amino acids such as phenylalanine) and carboxypeptidase B (CPB: recognizes C-terminal basic amino acids such as arginine)) by only changing the conjugated amino acid [108]. Since hippuryl amino acid derivatives are known substrates for CPs their aliphatic carboxylate part was incorporated into the rhodamine at its amines [113,114]. 

**Figure 4:**
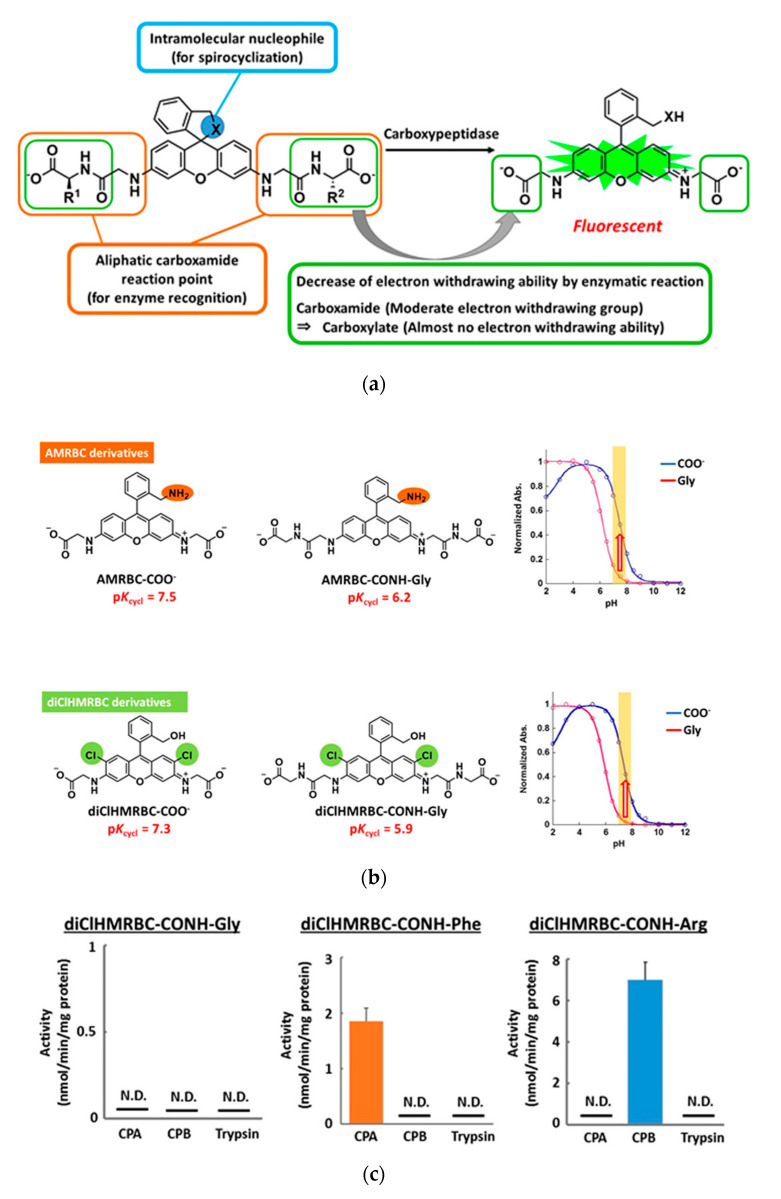
Novel probes for the detection of carboxypeptidases: (**a**) Molecular design of an activatable fluorescent probe for CPs; (**b**) AMRBC and diClHMRBC analogues and the change in pK_cycl_; (**c**) Activation of **diClHRBC-CONH-Gly**, **diClHMRBC-CONH-Phe**, or **diClHMRBC-CONH-Arg** with CPA, CPB or trypsin. Adapted with permission from [107]. Copyright (2018) American Chemical Society.

Previously, we observed that the electron-donating or -withdrawing ability of the alkyl substituent on the nitrogen atoms of the xanthene moieties effects the pK_cycl_ value [115,116]. This in mind, a carboxamide (an amino acid substrate bound to the probe) has a higher electron-withdrawing affinity than the corresponding carboxylate (when the substrate was cleaved by the CP) leading to a change in its pK_cycl_ value (Figure 4a). Even though the predicted effect was observed, the pK_cycl_ values still did not render the probes useful at the desired physiological pH. To further optimize (lower) the pK_cycl_ values of the probe aminomethyl rhodamine AMRBC (higher nucleophilicity of the intramolecular nucleophile) and dichloro hydroxymethyl rhodamine diClHMRBC (the chlorines lower the LUMO level of the xanthene) analogues were synthesized (Figure 4b). These probes were coupled to phenylalanine (Phe) and arginine (Arg) respectively, to target the two CPs (Figure 4c). The diClHMRBC probes showed higher activation ratios due to their lower pK_cycl_ values and depending on the attached amino acids CPA (diClHMRBC-CONH-Phe) and CPB (diClHMRBC-CONH-Arg) were successfully detected (Figure 4c). The probes also worked in pancreatic juice, which might be useful to detect pancreatic fistula, one of the most serious complications after digestive surgery and which can cause death [117]. The control probe diClHMRBC-CONH-Gly coupled to glycine showed no activity with either CP.

**Figure 5:**
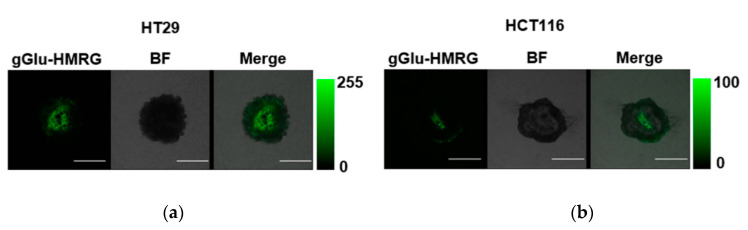
Fluorescence imaging of **gGlu-HMRG**: in HT29 (**a**) and HCT116 (**b**) tumor spheroids. Adapted with permission from [118]. Scale bars = 500 µm.

Our group recently also reported that a previously published gGlu-HMRG probe that targets γ-glutamyltranspeptidase (GGT) can rapidly detect metastatic lymph nodes (mLN) of colorectal cancer (CRC) [118]. Even though we previously showed that the probe cannot effectively detect primary human CRC, the rapid detection in lymph nodes was possible [119]. The reason for this is an induction of high levels of GGT because of hypoxia and low nutritional status due to insufficient blood supply. We detected high expression of GGT in mouse models and human CRC cell lines HT29 and HCT116 (Figure 5).

**Figure 6:**
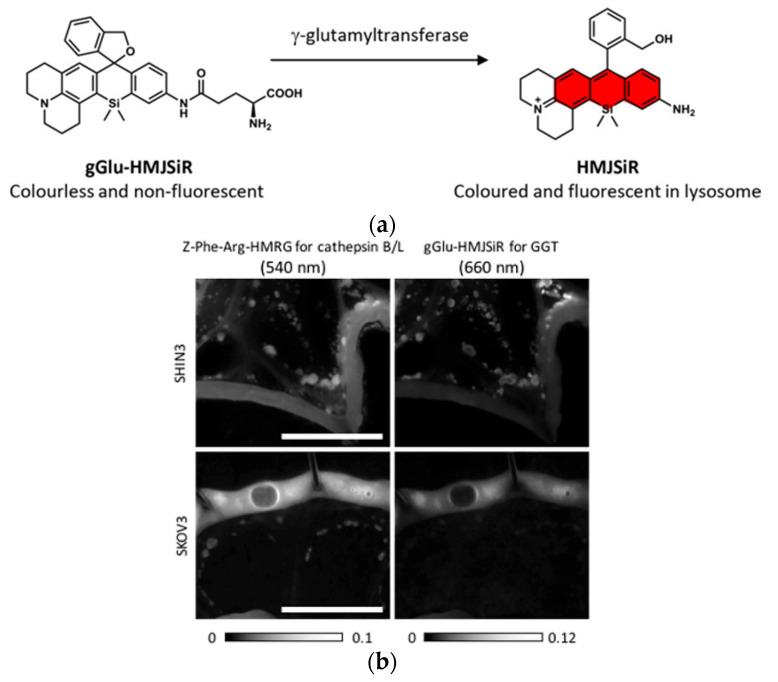
Si-rhodamine near-infrared probe for the detection of GGT: (**a**) Structure of **gGlu-HMJSiR** and its fluorescent **HMJSiR** reaction product; (**b**) Ex vivo fluorescence imaging of mouse models with peritoneal metastasis of SHIN3 (top) or SKOV3 (bottom) cells, 60 min after intraperitoneal application of Z-Phe-Arg-HMRG and topical application of **gGlu-HMJSiR**. Adapted with permission from [120]. Copyright (2018) American Chemical Society. Scale bars = 1 cm.

In a similar fashion we could show that the same probe is suitable to detect thyroid carcinoma in resected human samples [120]. Since gGlu-HMRG seems a promising cancer related GGT detecting probe it would be beneficial if more colors were available, especially in the NIR region of the spectrum. We recently developed a Si-Rhodamine near-infrared probe gGlu-HMJSiR based on HMJSiR (λ_exc_/λ_em_ = 637 nm/662 nm, compared to HMRG with 501 nm/524 nm) which shows a strong increase in fluorescence (145-fold at pH 7.4) just minutes after addition of GGT (Figure 6a) [121]. A dual-color experiment ex vivo with gGlu-HMJSiR (targets GGT) and Z-Phe-Arg-HMRG (targets cathepsin B/L) in mouse models of injected SHIN3 cells (exhibit high activities of both GGT and cathepsins) and SKOV-3 cells (low activity for GGT but high activity for cathepsins) was performed (Figure 6b). We could show a green fluorescence signal emerging in both SHIN3 and SKOV-3 nodules but the NIR fluorescence signal of HMJSiR was only observed in SHIN3 nodules. 

**Figure 7:**
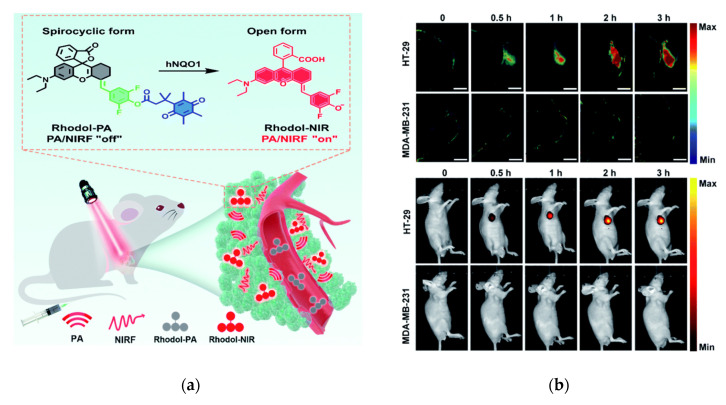
Cancer targeting photoacoustic probe: (**a**) Illustration of the activation mechanism of **Rhodol-PA** for PA/NIRF imaging; (**b**) Representative photoacoustic (**top**) and fluorescence (**bottom**) images of HT-29 and MDA-MB-231 tumor-bearing living mice at different time points upon intratumoral injection of **Rhodol-PA**. Adapted with permission from [130]—Published by The Royal Society of Chemistry. Scale bars = 2 mm.

Photo acoustic (PA) imaging has started to become a powerful biomedical imaging tool that enables non-invasive visualization of biological processes at molecular and cellular levels in deep tissue with high spatial resolution [122,123,124]. Penetration depth of a few centimeters can be achieved using the near-infrared (NIR) operating window and it has been used for many interesting applications [125,126]. The most common probes are based on intramolecular charge transfer (ICT) and the contact quenching mechanism [127,128,129]. Nevertheless, these probes show only limited imaging contrast due to small shifts in their absorption spectra or quantum yields. The group of Jiang succeeded in rationally designing a NIR rhodol dye for high contrast photo acoustic imaging [130]. Their spirocyclic dye Rhodol-PA with a non-aromatic cyclohexene ring is coupled to a dichlorophenol moiety which can be bound to a substrate via an ester bond (Figure 7a). Upon cleavage of that ester bond the uncaged fluorophore (λ_exc_ = 700 nm, λ_em_ = 720 nm) shows a large extinction coefficient, superb photostability and low quantum yield (which is important in PA imaging, as a low quantum yield indicates a high efficiency in photo-to-thermal conversion). Its efficacy was tested in vitro and in vivo to image hNQO1 activity, an important cancer-related biomarker, via PA/NIRF dual-mode detection. With MDA-MB-231 (cancer with a low hNQO1 expression) and HT-29 (high hNQO1 expression) cells xenografted live mice showed that in MDA-MB-231 tumor bearing mice negligible response was detected, whereas HT-29 bearing mice expressed a strong increase in signal (Figure 7b). High signal-to-noise, high sensitivity, and high selectivity were achieved. 

### 2.2. Organelle and Protein Labeling

The visualization of organelles plays an important role in biological sciences. Each organelle contains its own proteome, which is involved in its structural and functional properties [131]. Lysosomes, often referred to as the stomach of the cell, mitochondria, the powerhouse of the cell, and the cell nucleus are just some of the important organelles in cells. The targeting of specific organelles is of great interest; Mitochondria for instance play a critical role in several vital processes such as ATP production, central metabolism and apoptosis and a dysfunction is related to many diseases [132]. 

**Figure 8:**
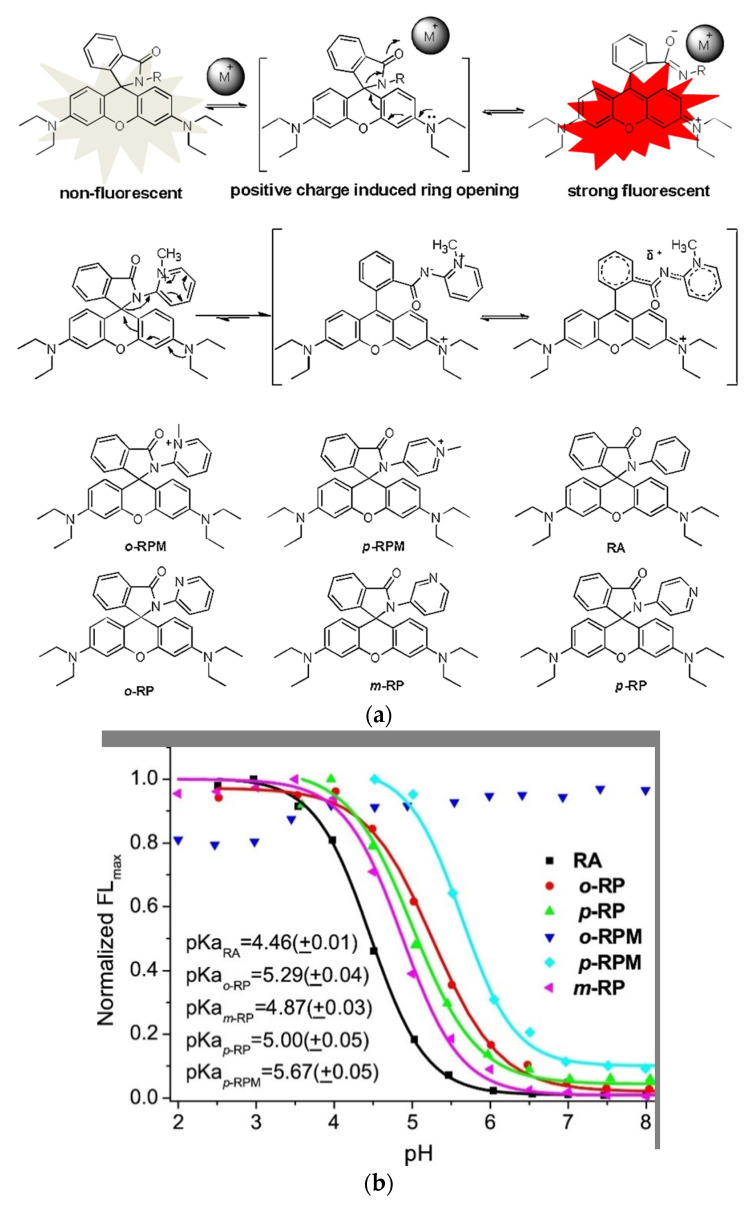
Acid resistant, always-on, mitochondria targeting probe: (**a**) (**top**) Metal ion induced ring-opening reaction of rhodamine spirolactam; (**middle**) Dominant structure of “always-on” rhodamine spirolactam **o-RPM**; (**bottom**) Structures of **o-RPM** and some rhodamine control spirolactams; (**b**) Normalized intensity at emission maximum vs various pH values in aqueous solutions of **o-RPM** and controls. Adapted with permission from [133].

To target mitochondria and simultaneously produce a water-soluble always on probe Xiao and coworkers mimicked the spirolactam opening effect induced by metal-ions (Figure 8a, top) [133]. They incorporated a positive charge via a quarterly o-aminopyridine, that resembles the positive charge induced by bound metals (Figure 8a, middle) [134]. The probe o-RPM shows florescence at a broad pH range (pH 3.5–13.0) and also in aprotic solvents such as DCM, DMSO or acetonitrile. A pH titration of o-RPM and a range of similar control molecules (Figure 8a, bottom) showed that the controls were quenched at higher pH-values (pK_cycl_ 4.46–5.67), whereas the target molecule showed excellent stability over a broad pH range (Figure 8b). Mitochondria selectivity was confirmed by co-staining with Rhodamine 123, a commercial mitochondrion tracker.

**Figure 9:**
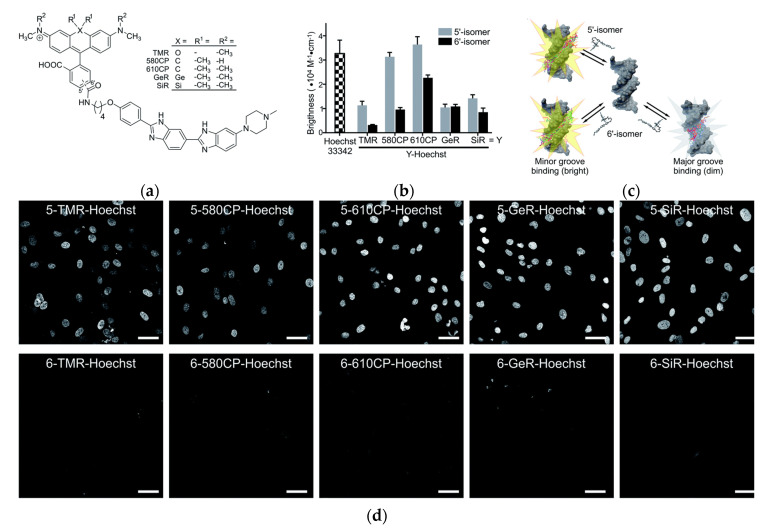
Novel dyes for the staining of chromatin: (**a**) Structures of the Hoechst derivatives; (**b**) the brightness after binding to specific DNA; (**c**) the proposed model of rhodamine–Hoechst conjugate interaction with the target DNA; (**d**) Representative confocal images of human fibroblasts stained with different 5′- and 6′-Hoechst dyes. Adapted with permission from [135]—Published by The Royal Society of Chemistry. Scale bars = 50 µm.

An interesting observation about the staining of chromatin was observed by the Lukinaviĉius and Hell group [135]. Chromatin is a complex structure which is made up from proteins, DNA and RNA. Fluorescence microscopy of chromatin in living cells is used to investigate cell division, apoptosis, necrosis, and other crucial events [136,137,138,139]. Hoechst and DAPI (4′,6′-diamino-2-phenylindole) dyes are commercially available and usually used to dye said complex structure in the cell’s nucleus [140,141]. The newest cell-permeable DNA probe is SiR-Hoechst (SiR-DNA) which allows imaging of the cell’s nucleus in the near-infrared region [142,143]. SiR-COOH, which SiR-Hoechst is based on, tends to exist in its spirocyclic form before binding to proteins, but can be converted to the fluorescent xanthene form after binding. These and related fluorophores can either exist as 5′- or 6′-carboxy-isomers and only recently the 4′-carboxy-isomers were synthesized by the same group, which will be addressed at a later point in the review [144,145,146,147,148]. Depending on the location of the carboxylic acid, the spectral properties might be identical, but their localization and cytotoxicity can be different. So far there have only been a few reports that investigated the performance of the different regioisomers in detail [149,150]. The group synthesized 5 different Hoechst dyes in both (5′ and 6′) configurations and with different heteroatoms (O, C, Si and Ge) that target chromatin and compared their considerable different complexations with the target DNA (Figure 9a). Eventually they found that the 5′-regioisomer is superior to the 6′-regiosisomer, as it yielded up to 10-fold brighter nuclear staining, probably due to a different localization on the target DNA (Figure 9b,c). Confocal images of human fibroblasts stained with the dyes showed the superior staining by the 5′-isomers (Figure 9d). While the 5′-regioisomers seem to have a higher affinity towards binding the minor groove of DNA, the 6′-regioisomers can also interact and bind to the major groove, which yields a dimmer complex. This tendency could be confirmed by docking and titration experiments.

**Figure 10:**
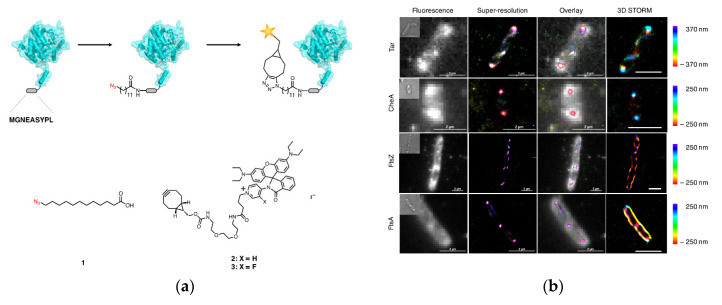
Protein labeling strategy: (**a**) A bacterial protein of interest is outfitted with a short N-terminal nonapeptide NMT recognition sequence (MGNEASYPL). Treatment of cells with **1** during the expression of the target protein results in the azide-labeled protein. Azide−alkyne cycloaddition with cell-permeant photo switchable dyes such as **2** and **3** tags the protein of interest for super-resolution imaging; (**b**) STORM images of bacterial proteins with polar localization (Tar and CheA) or septal localization (FtsZ and FtsA) expressed in *E. coli*. Adapted with permission from [152]. Further permissions related to the material excerpted should be directed to the ACS. Scale bars = 2 µm.

Not only organelles are important staining targets, but also enzymes and proteins in general [151]. A novel way to enzymatically label bacterial proteins for super resolution imaging in living cells was developed by Tirrell and coworkers [152]. Many dyes used for super resolution imaging require oxygen-scavenging buffers, exogeneous reducing agents, or the specimens to be fixed or permeabilized to label intracellular targets [153,154,155]. The group recently reported a general strategy for the chemoenzymatic labeling of bacterial protein with azide-bearing fatty acids in living cells by the eukaryotic enzyme N-myristoyltransferase (NMT) [156,157]. This strategy was used to label proteins (FtsZ, FTsA, Tar and CheA) in live E. coli with cell-permeant bicyclonyne-functionalized rhodamine spirolactams [158]. Rhodamine spirolactams can be present in either the non-fluorescent “closed” form or the highly fluorescent, colored “open” xanthylium form. Absorption of UV light can break the bond between the lactam nitrogen and the xanthene ring, restoring the conjugation in the xanthene ring. This open isomer emits photons until either thermally reverting to the more stable dark isomer or photobleaching [159,160]. The proteins of interest were outfitted with a nonapeptide sequence MGNEASYPL, which serves as an N-terminal NMT recognition sequence derived from mammalian protein calcineurin B (Figure 10a) [161]. After expressing the proteins of interest (chemotaxis proteins CheA and Tar, cell division proteins FTsZ and FTsA) by activating the araBAD promotor with L-arabinose, N-terminal labeling was achieved with the addition of 1 (12-azidododecanoic acid, 12-ADA, Figure 10a). After incubation, the labeling fluorophores 2 and 3 were added and super resolution imaging (STORM) in live E. coli was performed (Figure 10b). While CheA and Tar were mostly clustered at the cellular poles, FtsZ and FTsA were found to be mostly localized near the septum of the cell. Reconstructed images furthermore revealed smaller clusters of Tar throughout the cell and even banded or helical patterns. The high-resolution images of FtsZ also showed elongated clusters of variable orientation throughout the cell.

**Figure 11:**
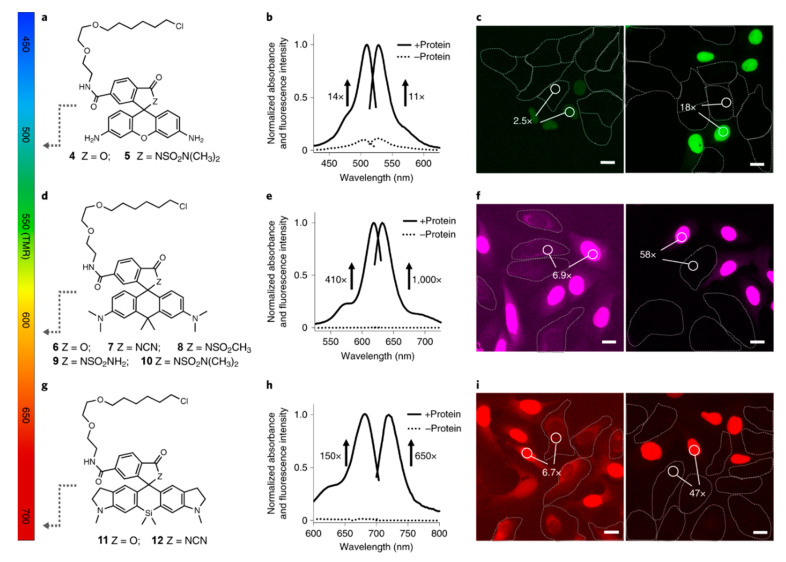
Cell-permeable probes with wavelengths that range from cyan to the near-infrared for no-wash live-cell microscopy; (**a**,**d**,**g**) Structures of R11, CPy and SiR700 derivatives coupled to HaloTag substrate, (**b**,**e**,**h**) Respective absorption and emission spectra of **5**, **10** and **12**; (**c**,**f**,**i**) No-wash live-cell confocal images of co-cultured normal U2OS cells and U2OS FlpIn Halo-SNaP- NLS-expressing cells labelled with **4**, **6** and **11** (**left**) and **5**, **10** and **12** (**right**). Adapted by permission from Nature: Springer, Nature Chemistry [170] Copyright (2019). Scale bars = 20 µm.

Many synthetic small molecular fluorophores have been developed, yet it is still challenging for most fluorophores to show good cell permeability due to their often-charged properties [162,163]. These probes can only be used in live cell imaging if invasive techniques like permeabilization, squeezing, bead loading or microinjections are used to force the fluorophore into the cell [164,165,166]. Spirocyclic xanthene-based probes, when in their hydrophobic, spirocyclic non-charged form usually show good cell-permeability. However, most of the used probes exist in the form of the zwitterionic structures and thus exhibit a lower cell permeability. One way to synthetically force the probes to take the spirocyclic structure is to introduce electron-withdrawing groups (EWGs) on the xanthene core [52,142,167]. This structural change, however, can lead to changes in their spectroscopic behavior like reduction of quantum yield or increase in susceptibility towards reactions with nucleophiles [167,168,169]. The group of Johnsson developed a strategy to increase the cell permeability of fluorescent probes without changing their spectroscopic properties [170]. It is known that when the carboxylic acid responsible for the formation of the spirolactam form is exchanged to an amide the closed form is preferred, even after binding to their targets [19,134]. They destabilized the spirolactam by attaching different EWGs to the nitrogen atom of the amide of 6-carboxyetetramethylrhodamine (6-TAMRA), a widely used fluorophore. The absorbances of the parent probe and four amide probes (cyanamide, acyl sulfonamide and two acyl sulfamides) were measured at different dielectric constants. All amides showed similar spectroscopic properties, but their D_50_-values changed from 15 (parent molecule) to 32–60 indicating their potential to be cell permeable. The D_50_-value indicates at which dielectric constant the absorbance of a dye is halved. The group used their enhanced probes to visualize different targets in live-cell no-wash microscopy by binding them to different tags (SNAP-Tag, HaloTag, jasplakinolide and docetaxel). The HaloTag-probes 5, 7, 8, 9, 10, and 12, for example, showed superior staining properties compared to their parent probe analogues 4, 6, and 11 (Figure 11). Since multi-color imaging is favorable the group also synthesized different silicon- and carbon-bridged analogues and validated their superior cell permeability and higher signal-to-noise ratios through microscopy and STED microscopy.

### 2.3. Therapy and Diagnostics

**Figure 12:**
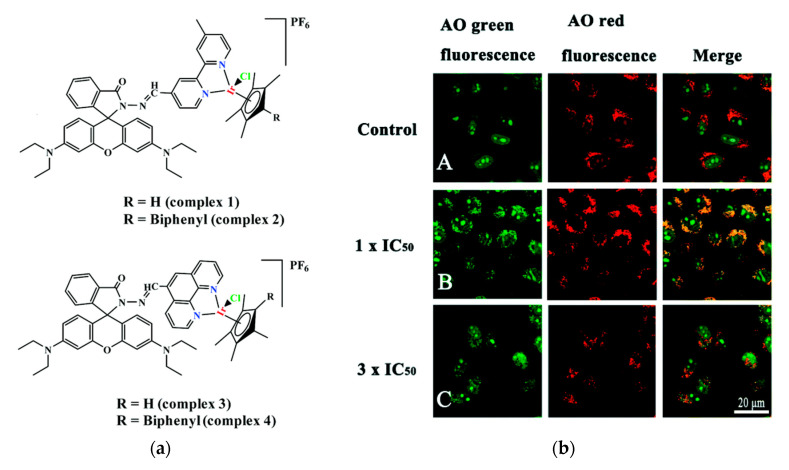
Ir-piano-stool anticancer drugs: (**a**) Structures of four iridium piano-stool rhodamine anticancer drugs; (**b**) Lysosomal membrane permeabilization in A549 cancer cells. A549 cells without (**A**), with 1 × IC_50_ (**B**) and 3 × IC_50_ (**C**) **complex 3** and then stained with AO. Adapted with permission from [189].

Cancer is one of the major diseases that humanity is facing. Many therapies to cure or slow down its development have been developed and many of them include small molecule drugs [171,172]. One major issue with these drugs is that they tend to attack healthy cells as well. The most widely used anticancer drug is Cisplatin, which is also the only metal-based anticancer drug used clinically [173,174,175]. Even though it is widely used, it has toxic side effects, which led researchers to develop non-platinum metal anticancer drugs [176,177,178]. Nowadays several other transition metal complexes like iridium complexes that show anticancer activity have been developed [179,180,181,182,183,184]. However, half-sandwich iridium complexes for example suffer from unknown targets, unclear mechanisms and poor selectivity between cancer and normal cells [185,186]. One target of anti-cancer compounds are lysosomes, which are evolutionary conserved organelles that are thought to play a big role in the regulation of apoptosis [187,188]. To better understand and address the above-mentioned issues the group of Liu developed four rhodamine coordinated iridium complexes complex 1–complex 4 which show high anticancer activity (Figure 12a) [189]. 

Their complexes were bound to rhodamine B which allowed the complexes to be fluorescent while simultaneously enhancing their anticancer activity. The observation of an improvement of a compound’s anticancer activity by binding it to rhodamine B was also recently confirmed by Dehelean and coworkers [190]. They increased the antitumor activity of oleanolic acid by bioconjugating it to rhodamine B. This bioconjugated mitocan (agents that directly target and alter the function of mitochondria in cancer cells leading to cancer cell growth inhibition or apoptosis) resulted in better solubility of the compound and due to the possibility of rhodamine B to take the spirocyclic form, cell membrane passage was facilitated. For example, Lui’s complex 3, which enters the lysosome via an energy-dependent process showed apoptotic activity in A549 cells, superior to cisplatin (Figure 12b). Experiments with circulating-tumor DNA (ctDNA) showed that the drug’s target is not DNA. Instead, a high affinity to the tryptophane microenvironment of bovine serum albumin (BSA), a widely used human serum albumin (HSA) substitute, was detected. The drug accumulated in the lysosomes, which causes a change in the osmotic pressure, leading to lysosomal membrane permeabilization (LMP) and hence apoptosis by infiltration of lysosomal proteases. The apoptotic cells could be visualized by staining with acridine orange (AO). This metachromatic fluorophore is retained within the lysosome and emits red fluorescence when charged (AOH^+^). After drug induced apoptosis the lysosomes are damaged, and the dye leaks out and becomes non-fluorescent. The group also reported 12 other novel fluorescent iridium (Ir) and ruthenium (Ru) complexes which bear similarities in structure and reactivity to the above mentioned [191,192]. 

Another possibility to target cancer is photodynamic therapy (PDT) which is also used as a treatment for other diseases [193]. This minimally invasive method uses photosensitizers (PS) and light irradiation to produce reactive oxygen species (ROS) like singlet oxygen (^1^O_2_) to induce cell apoptosis [194]. Even though this kind of therapy is less invasive and can be repeated with minimal side effects, conventional PDT agents only show poor tumor selectivity and thus PDT treatment may cause severe damage to healthy tissue [195,196,197]. Currently employed Se-rhodamines, for example, are showing good properties for utilization in PDT but suffer from an activation by green light which lies outside of the therapeutic window (600–900 nm) [198,199,200,201]. The Liu group designed a Se-rhodamine scaffold with an extended π-conjugated system that yielded in red-absorbing photosensitizers [202]. 

**Figure 13:**
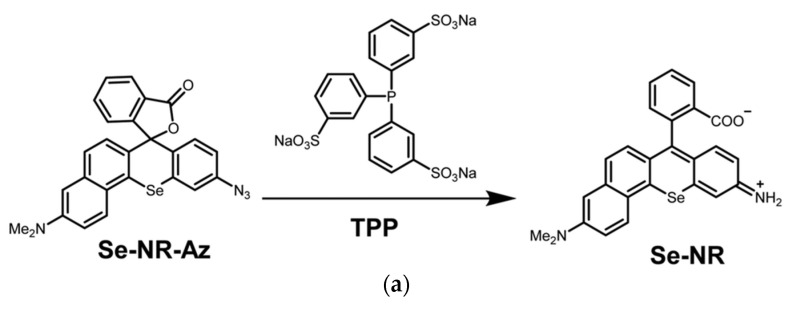

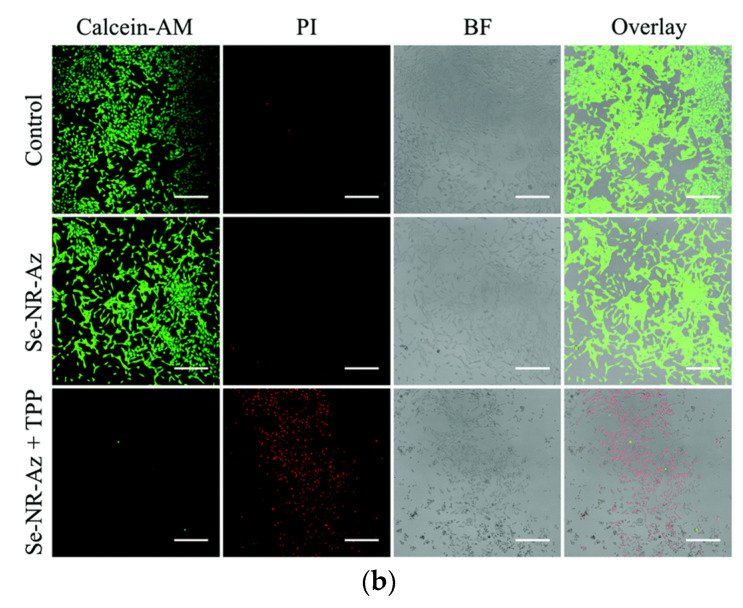
Bio-orthogonally activatable photosensitizer by Staudinger reaction: (**a**) Activation strategy and chemical structures of **Se-NR-Az** and its decaged product **Se-NR**; (**b**) Confocal images of calcein-AM and PI stained HeLa cells without, with **Se-NR-Az** and with **Se-NR-Az** + **TPP** different treatments. Adapted with permission from [193]. Scale bars = 240 µm.

In contrast to the common activation of fluorophores and photosensitizers by overexpressed enzymes they chose the Staudinger reaction (a reaction of a phosphine like TPP with an azide yielding an amine in aqueous solution) as a biorthogonal activating method, which has already been employed for drug release before (Figure 13a) [203,204,205,206,207]. The masked rhodamine Se-NR-Az shows only little visible absorption and cannot produce singlet oxygen, whereas the demasked rhodamine Se-NR showed a production of significant amount of ^1^O_2_ (which was determined by reaction with the singlet oxygen trap 1,3-diphenylisobenzofuran) at excitation at 630 nm. The photocytotoxicity was tested in HeLa cells in vivo with up to 200 mM triphenylphosphine (TPP) to yield an IC_50_ value of 0.205 mM which is comparable with clinical Protoporphyrin IX (PPIX, a photosensitizer used in the photodynamic therapy of cancer with IC_50_ = 0.229 mM). The cells were stained with calcein-AM (calcein acetoxymethyl, a cell permeable fluorophore that is used to test cell viability with green fluorescence) and PI (propidium iodide, a red fluorophore that can stain DNA and is used to visualize necrotic and apoptotic cells, since it is not membrane-permeable) to show that only after addition of TPP the cells died (Figure 13) [208].

**Figure 14:**
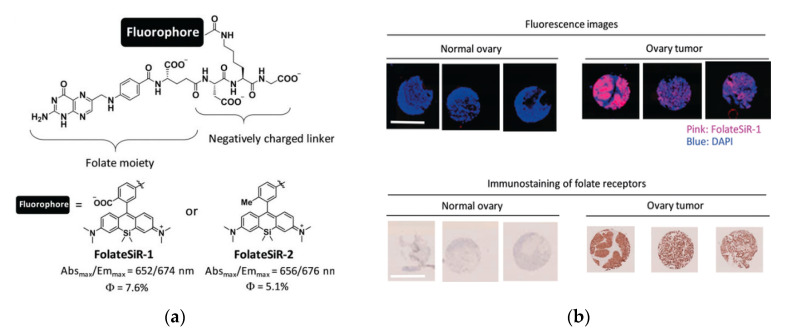
Immunostaining of folate-receptor expressing tumors: (**a**) Molecular design of fluorescent probes for detection of folate receptors and the structures of **FolateSiR-1** and **FolateSiR-2**; (**b**) Ovarian cancer tissue microarray with **FolateSiR-1** and DAPI (nuclear stain). Adapted with permission from [209]. Scale bars = 2 mm.

Our group developed a fluorescent probe that can rapidly visualize folate-receptor-expressing tumors with high contrast [209]. The alpha isoform of folate receptor (FR-α) is upregulated in 40% of human cancers, especially in malignant tissues such as ovarian cancer [210,211]. Normal tissues besides the kidney, on the other hand, do not accumulate folic acid or its conjugates [212,213,214]. Various folate-linked drugs and imaging agents have been developed and recently folate-FITC is being employed in intraoperative tumor specific screening in patients [215]. Nevertheless, its green emission (520 nm) is unsuitable for imaging deep tissues and existing dyes emitting in the NIR show nonspecific adsorption and require washing steps [216,217,218]. We were able to develop a FR-α-probes FolateSiR-1 and FolateSiR-2 that contain the folate moiety coupled to a Si-rhodamine by an Asp-Lys-Gly linker (Figure 14a). This negatively charged linker was employed to reduce the cell permeability of the probe, since the folate receptors are outside the tumor’s membrane. Folic acid binds to FR-α with a high affinity (K_d_ ≈ 10^−9^ M) and undergoes receptor-mediated endocytosis after binding [212]. With FolateSiR-1, we were able to visualize patient derived ovarian tumor tissues while exhibiting little binding to normal tissue in a microarray (Figure 14b). The fluorescence image of the tissue microarray and the immunostaining image matched well, and normal tissue did not show any increase in fluorescence.

### 2.4. Small Molecule Detection

Biothiols, for example, are important antioxidants during oxidative stress or injury, serve as chelators for metals and act as essential signaling molecules. For example, glutathione (GSH) the most abundant non-protein biothiol, is involved among others in the maintenance of intracellular redox activities and signal transduction, proliferation, apoptosis, and gene regulation [219,220]. Its concentration ranges intracellularly from 1–10 mM and extracellularly from about 5–25 µM. Abnormal levels of biothiols are linked to several diseases, including cancer. Cysteine (Cys) for example is linked to liver damage, slow growth, or edema, while homocysteine (Hcy) is associated with cardiovascular and Alzheimer’s disease, and H_2_S is linked to colorectal cancer [221,222,223]. Given their importance in cell metabolism fluorescent probes have been developed to track the concentration of biothiols in real time with high sensitivity and selectivity [224]. 

**Figure 15:**
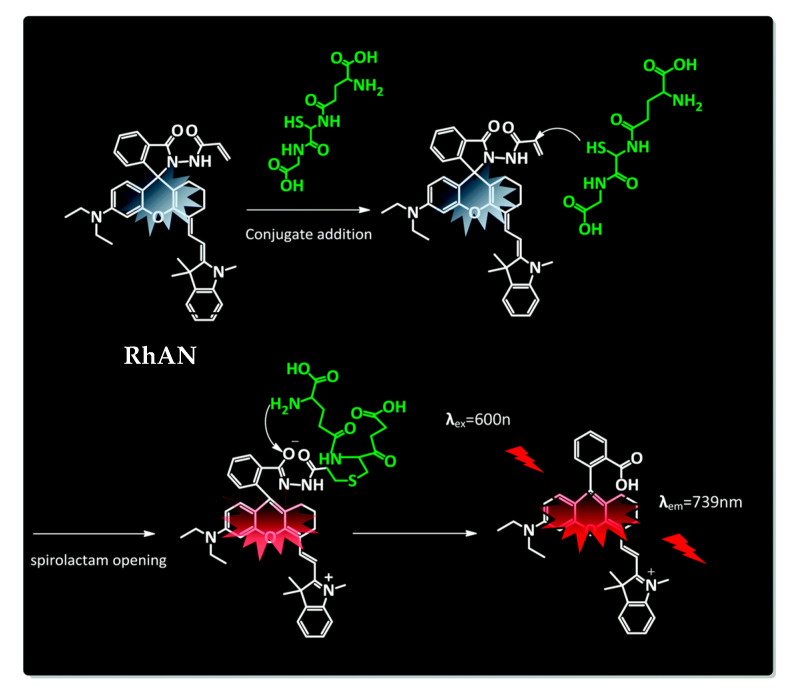
The possible mechanism of **RhAN** sensing toward GSH based on conjugate addition and intramolecular amino induced spirolactam opening. Adapted with permission from [225].

However, that most probes lack discrimination between the different biothiols still presents a challenge. A novel glutathione selective near-infrared probe was developed by Quian and coworkers that could detect GSH over Cys and Hcy within 5 s by the naked eye [225]. Their system is based on a conjugate addition by a Knoevenagel reaction and intramolecular amino induced spirolactam opening (Figure 15). The chemosensor RhAN absorbs at 717 nm and emits at 739 nm with an increase of fluorescence of 90-fold after addition of GSH (20 mM), while other amino acids showed almost no increase in fluorescence. 

**Figure 16:**
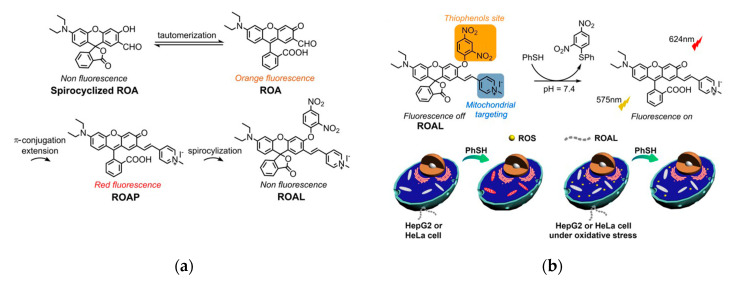
Detection of mitochondrial thiophenols: (**a**) Structures of the probes **ROA**, **ROAP** and **ROAL**; (**b**) The proposed sensing mechanism of **ROAL** towards thiophenols. Adapted with permission from [226].

The group of Guo tried to visualize mitochondrial thiophenols and their induced oxidative stress process in live-cells, via a rationally designed rhodol-based probe [226]. Thiophenols are among the most poisonous compounds among environmental toxic substances [227,228]. Since mitochondria are the preferential targets for thiophenols, they are linked to pathogenic ROS production [229]. Many probes have been developed, but none was able to detect thiophenol, specifically in mitochondria [230,231,232]. Their probe ROAL makes use of their previously synthesized rhodol dye ROA which showed high quantum yields (Φ = 0.94, in ethanol) and stability in aqueous solution (Figure 16a) [233]. To target mitochondria, a pyridinium cation and to sense thiophenol, a 2,4-dinitrophenyl group were introduced into the molecule to give ROAL. While ROAL is non-fluorescent the product after reaction with thiophenol ROAP is strongly fluorescent (Figure 16b). The modification by introduction of the pyridinium cation to ROA led to a red shifting in its excitation wavelengths by 60 nm, from 515 nm to 575 nm. A selective detection of thiophenol and selective targeting of mitochondria was achieved. Furthermore, they could show for the first time that trace amounts of thiophenols can be eliminated by endogenous ROS in living HePG2 and HeLa cells.

**Figure 17:**
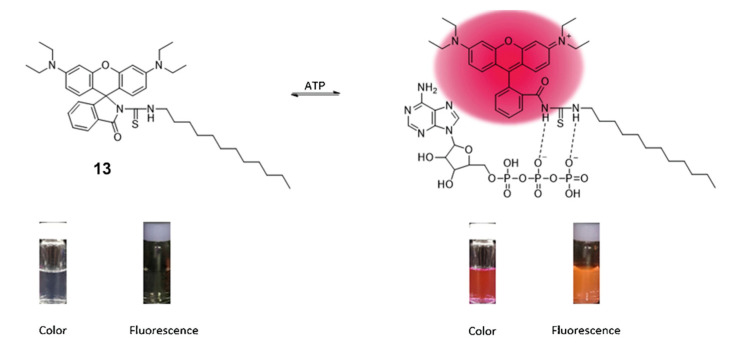
The colorimetric and fluorescent change of the mitochondria probe **13** with ATP by naked eye and its proposed binding mechanism. Adapted with permission from [234].

The group of Yoon and coworkers developed a way to visualize ATP in lysosomes and mitochondria [234]. ATP is among the most important substances for the existence of life as it functions as an energy source in all living organisms [235]. Besides its essential role as an energy source, it also plays a role in many important processes such as DNA synthesis, cell division, neurotransmission, and ion channel function. Low levels of ATP can be a sign of Parkinson’s disease, cardiovascular disease, or ischemia [236]. Because of its important role on living organisms many probes have been developed to target ATP [237,238,239,240,241]. Most of them rely on electrostatic or hydrogen bonding interactions with the negatively charged trisphosphate group of ATP. Also, boronic ester formation with hydroxyl groups of the ribose ring and π-π interactions with its adenine base were utilized [239]. The group synthesized two probes that contain a thiourea group which, upon reaction with the nucleoside’s triphosphate, forms the colorful open species (Figure 17). To selectively target mitochondria a dodecanoyl group was incorporated, whereas to target lysosomes a morpholine group was introduced [242,243]. A screening of biologically relevant biomolecules resulted in no significant signal, only ADP showed a 30% fluorescence response compared to ATP for both probes. Lysosome selectivity was moderate whereas the mitochondria-probe localizes specifically in mitochondria, showing only low cytotoxicity.

**Figure 18:**
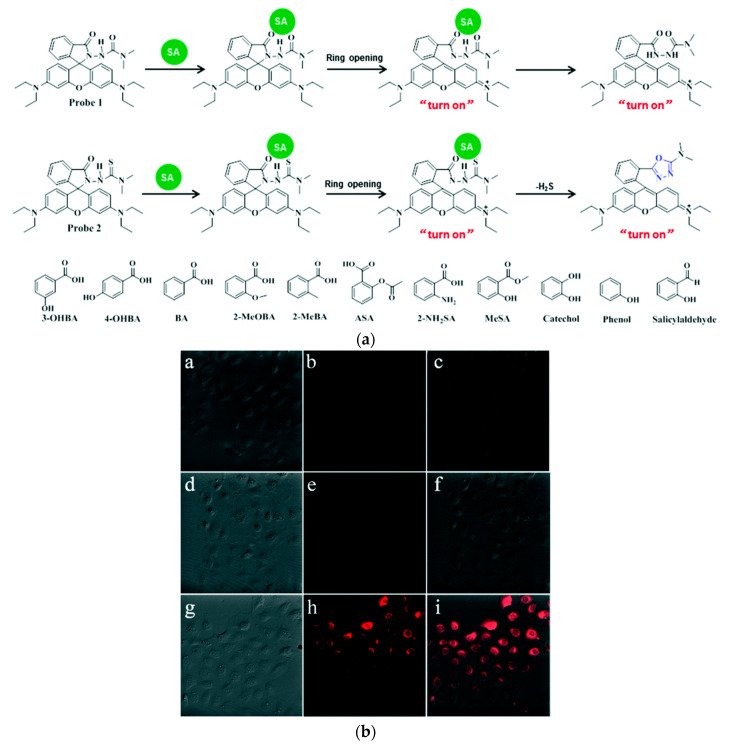
Detection of salicylic acid: (**a**) Proposed mechanism for detecting SA using probe 1 (**top**) and 2 (**middle**) and structures of tested SA analogues (**bottom**); (**b**) bright-field (**left**) and fluorescence (**middle**) and merged (**right**) images of NRK-52E cells; control (**top**) **probe 2** (**middle**) and **probe 2** + SA (**bottom**). Adapted with permission from [244].

A probe for the detection of the biologically signaling molecule salicylic acid (SA) was developed by Yang and coworkers [244]. SA is an important phytohormone and plays a role in the immune response against pathogens [245,246]. It can trigger global transcriptional reprogramming (global changes in gene expression that are typically initiated by transcription factors) and induce plant systemic expression of the resistant pathogenesis-related species [247]. Some fluorescent probes detecting SA have been developed, yet they cannot be used in situ or in vivo [248,249]. The two newly developed probes probe 1 and probe 2 are structurally identical and just differ in an oxygen atom of the carbonyl group being exchanged to a sulfur atom (Figure 18a). 

Both probes showed excellent substrate specificity and even similar SA analogues do not increase the fluorescence. While the substrate specificity is the same, probe 2 showed much stronger absorption and emission behavior, as well as a much higher quantum yield (0.41 compared to 0.12). Their assumption is that the sulfur containing probe forms a 3,4-oxadiazole ring after binding to SA which changes the electronic properties of the molecule and thus the quantum yield. The viability of SA detection was also verified in NRK-52E cells in vivo with added 50 mM SA (Figure 18b). Furthermore, SA could be detected with a low detection limit of 2 nM.

**Figure 19:**
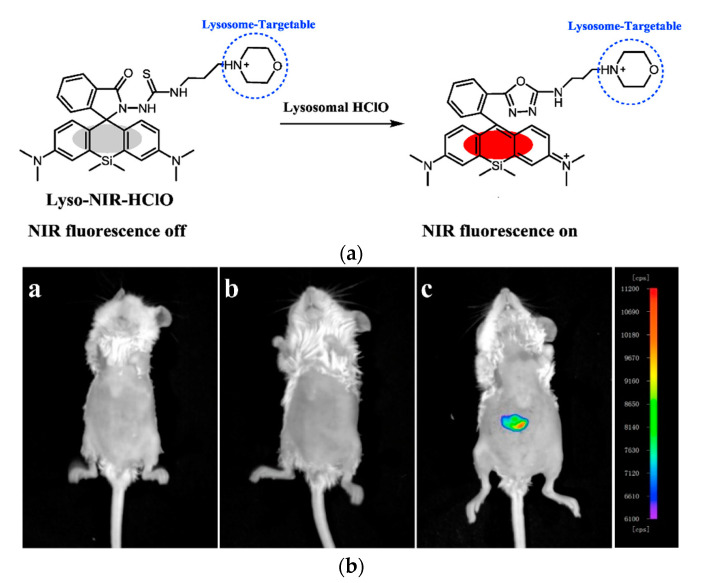
Detection of HClO in lysosomes: (**a**) The response mechanism of **Lyso-NIR-HClO** to lysosomal HClO; (**b**) Fluorescent images of in vivo HClO production from the peritoneal cavity of the mice with **Lyso-NIR-HClO** in inflamed mouse model: (**a**) negative control (**b**) **Lyso-NIR-HClO**; (**c**) **Lyso-NIR- HClO**, LPS and PMA. Adapted with permission from [250].

Interestingly, another structurally very similar probe was reported by Zhang and coworkers but for the detection of hypochlorous acid (HClO) [250]. The group successfully utilized the probe Lyso-NIR-HClO that targets lysosomes and emits in the near-infrared (λ_ex,max_ = 680 nm) to selectively detect HClO in vivo in an inflamed mouse model after activation with lipopolysaccharide (LPS) and phorbol myristate acetate (PMA) (Figure 19). The substrate specificity was tested with various biologically related substances and showed no interactions with other substrates. In this context SA was not tested as a possible target and vice versa HClO was also not tested as a substrate in the aforementioned probe (Figure 18). One should be aware of the similarity of the probes and possible false positive results.

### 2.5. Super-Resolution Microscopy

Super-resolution microscopy, developed by Betzig, Hell and Moerner was awarded the Nobel Prize in 2014, and became a powerful tool to visualize cells [251,252,253]. Several recent techniques in super-resolution imaging made it possible to look beyond the diffraction-limit of about ≈200 nm in common microscopy. The developed techniques include stimulated emission depletion (STED) [254,255], photoactivated localization (PALM) [256,257], and stochastic optical reconstruction (STORM) [258,259]. These have been widely used to image biomacromolecules or subcellular organelles with sub-diffraction resolutions. The principle of super-resolution PALM imaging for example makes use of “dark” and “fluorescent” pair states of the fluorescent dyes. Due to a quick change between the open and closed form a blinking is observed, which enables the temporal and spatial separation of adjacent molecules. While photoactivatable fluorescent proteins and small molecular fluorophores are employed, rhodamines remain the main fluorophore used, due to their superior photostability. The group of Yang developed a nitroso-caged photoactivatable rhodamine for PALM super-resolution imaging of lysosomes [260]. Even though a variety of photo-cages have been developed, only o-nitrobenzyl groups and a few others are being utilized as PALM probes [261,262,263,264,265]. 

**Figure 20:**
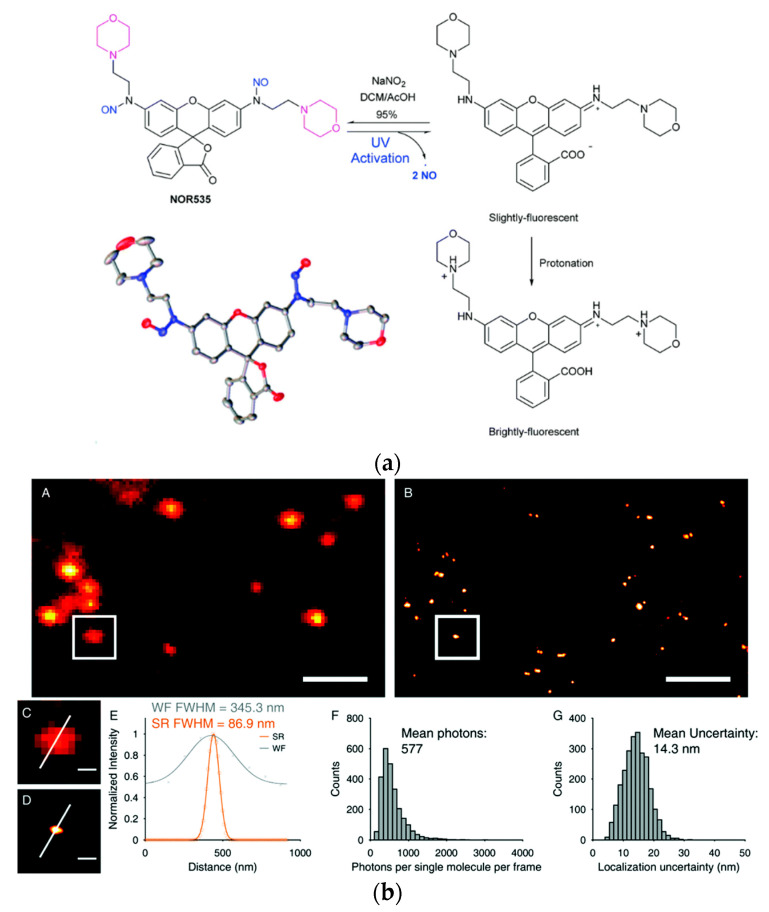
Biocompatible photo-caging strategy: (**a**) Structure, crystal structure and sensing mechanism of **NOR535**; (**b**) **NOR535** super-resolution imaging of lysosomes in live HeLa cells. Wide-field image (**A**) and PALM image (**B**) of the lysosomes; (**C**,**D**) Enlarged maps of the boxed areas of images A and B, respectively, showing a single lysosome. (**E**) Transverse profile of a single lysosome along the white line in C and D. (**F**) Histograms of the number of photons per single-molecule event. (**G**) Histograms of the localization precision. Adapted with permission from [260]. Scale bars = 2 µm (**A**,**B**), 300 nm (**C**,**D**).

Dyes with photo cleavable o-nitrobenzyl groups, release toxic and highly colored o-nitrobenzaldehyde upon cleavage. The newly reported probe NOR535 on the other hand is non-fluorescent and upon irradiation with UV light (405 nm) only endogenous and biocompatible nitric oxide is produced (Figure 20a). Two morpholine moieties were introduced to target lysosomes. After the nitric oxide was cleaved the resulting probe showed slight fluorescence (λ_exc_ = 520 nm, λ_em_ = 550 nm, Φ = 0.09), probably due to a PET quenching effect of the morpholines. Upon protonation (as mentioned before lysosomes are acidic organelles), however, bright fluorescence with a blue-shifted excitation and emission wavelengths was obtained (λ_exc_ = 510 nm, λ_em_ = 535 nm, Φ = 0.97). The photo-activatable probe was utilized in HeLa cells and colocalization with Lysotracker Red confirmed the selectivity for lysosomes. Super resolution PALM imaging of lysosomes in HeLa cells by a total internal refraction fluorescence microscope (TIRFM) showed reconstructed super resolution images with a detailed lysosomal morphology (Figure 20b). The transverse profiles of a single lysosome revealed a width of 86.8 nm. The number of mean photons emitted was ≈577 with a good localization precision of 14.3 nm.

**Figure 21:**
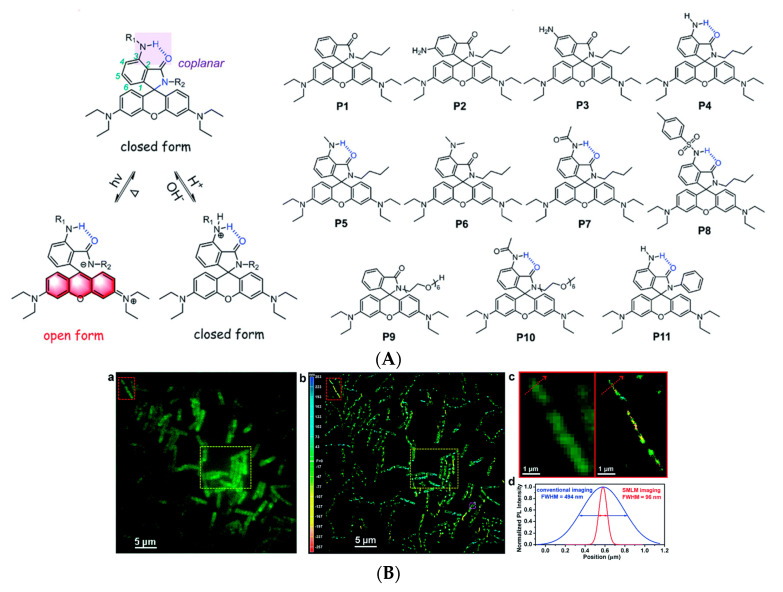
Photo-switchable, acid tolerant spirolactams: (**A**) Intramolecular hydrogen bonding enables acid-resistant photo-switchable rhodamine spirolactams and chemical structures of **P1–P11**; (**B**) (**a**) Conventional diffraction-limited fluorescence image and (**b**) 3D-SMLM image of live *Bacillus subtilis* cells stained with their final probe. (**c**) Comparison of the imaging resolution of the three adjacent *Bacillus subtilis* cells in the enlarged region, as highlighted by red rectangles. (**d**) Gaussian fittings to the normalized PL intensities across a randomly selected bacterium (cross-sectional analysis along the red arrow). Adapted with permission from [266]—Published by the Royal Society of Chemistry.

Another novel photo switchable rhodamine dye was reported by Xu and coworkers [266]. Their fluorophores are acid resistant spirolactams with a dedicated photo-switchable property (Figure 21(Ba)). 

Important for the acid-resistance behavior is the introduction of a 3-amino group in the carboxyphenyl ring **P4** which results in an intramolecular hydrogen bonding in spirolactams. Controls with the amino groups in different positions in the benzyl ring showed the importance of the 3-position for acid resistance. While the control molecules showed a band at 560 nm appearing in their absorption spectra during acidification, the 3-isomers showed only negligible sensitivity to TFA. The H-bond mechanism in these molecules was verified via NMR, crystallographic and density functional theory (DFT) experiments. The quantum yields were low, probably due to PET from the 3-aminophenyl moiety to the xanthene scaffold. Incorporating a 3-acetamido moiety **P7** increased the quantum yield from ≈0.05 to ≈0.3 while keeping the acid resistance. Introduction of polyethylene glycol (PEG) groups **P10** rendered the compound water soluble and a morpholine group made it possible to target lysosomes in MCF-7 cells. Furthermore, inspired by Moerner’s work on red-shifting the photoactivation wavelength by modifying the chromophore on the lactam nitrogen, the photoactivation wavelength was extended to the visible region (>400 nm) by conjugating the molecule with 6-phenylethynyl naphtalimide [158]. With their final probe bearing a N-hydroxysuccinimide for coupling purposes, they labeled the surface of acid-tolerant Gram-positive Bacillus subtilis cells in PBS (pH 4.5). By photoactivation with a 405 nm laser (1.8 W cm^−2^) and a 561 nm laser (1.2 kW cm^−2^) as an excitation source, excellent photo-blinking properties were found and reconstruction of the super-resolution image via 3D-SMLM was achieved (Figure 21(Bb)). The number of photons collected per 3000 frames (20 ms per frame) remained almost constant for >10 min rendering a potential probe for long time-lapse nanoscopy.

The group of Rivera-Fuentes, tried to overcome the problem of photobleaching, a phenomenon that is observed after prolonged excitation, by photo-regulation of fluxional fluorophores [267]. Fluxional molecules can undergo rapid degenerate rearrangements in the electronic ground state and exhibit stochastic switching behavior [268,269,270]. Classical photoactivatable dyes start from a dark isomer which transforms to the fluorescent state upon photoirradiation. The limitations of this method are phototoxicity and photobleaching after repetitive activation. 

**Figure 22:**
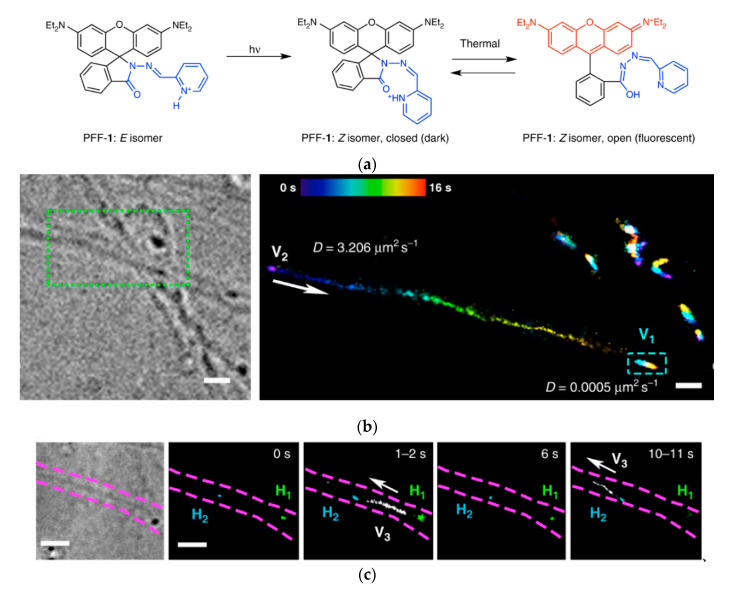
Fluxional fluorophores for less photobleaching: (**a**) Mechanism of photoactivation and fluxionality of **PFF-1**; (**b**) Bright-field image of a neuronal projection and synapse and an image reconstructed from all frames collected during a 16 s acquisition within the green ticked area, as well as the diffusion coefficients of two vesicles (V1 and V2); (**c**) Bright-field image and fast diffusion of an acidic vesicle (V3) between hotspots (H1 and H2) in 20 s of a single neuronal projection. Adapted with permission of [267]. Scale bars = 2 µm (**b**) and 1 µm (**c**).

Spontaneously blinking probes on the other hand exhibit a ground-state equilibrium between a dark and a fluorescent species which does not need photoactivation and thus results in a decrease in phototoxicity and photobleaching. These dyes are greatly dependent on the correct pH and polarity of the medium to exhibit their blinking correctly. However, imaging is only possible for short periods of time and long time-lapse imaging could only be achieved within the membranes low-polarity environment [42,47,271,272]. A combination of photoactivation and fluxionality can provide a method to control the number of molecules that are fluorescent independently from the medium. Starting from a dark, nonfluxional isomer, photoactivation then leads to the fluxional form. In the population of molecules in that form some exist in the dark and some in the bright state and their thermal equilibrium can be used for single-molecular imaging. Even after photobleaching of the dye, a new fraction can be activated, which enables extremely long time-lapse and single molecular acquisitions with minimal phototoxicity. This was achieved by incorporating a photo switchable acylhydrazone onto a rhodamine B scaffold to form PFF-1 (Figure 22a). This molecule exists predominantly as the E-isomer, which is a dark, nonfluxional, non-fluorescent spiro-compound. Photoisomerization with light of 410 nm led to a 12-fold and 22-fold increase in aqueous buffer at pH = 7 and pH = 5, respectively. A reference compound with a CH group in position 2 of the acylhydrazones aryl ring only showed 1.6-fold and 3-fold increases in the same buffers. Upon photoisomerization a fraction of the E-isomer changes into its Z-isomer, which does not revert thermally over a period of 2 h. In contrast to the E-isomer the Z-isomer can then thermally switch between the spirocyclic (dark) and open (fluorescent) form which absorbs strongly at 560 nm. At pH = 7.4 the overall fraction of fluorescent molecules could be estimated before and after photoactivation (410 nm, 100 s, ≈8.5 mWcm^−2^) as 0.003% and 0.012%. Due to the 2-pyridyl substituent being in close proximity to the carbonyl group, proton transfer-induced ring opening is facilitated, which was supported by DFT calculations. The barrier between the open fluorescent and the dark spirocyclic form was estimated to be 6.6 kcal mol^−1^ which is low enough to enable a rapid interconversion between these two states. Single molecular imaging showed a robust switching behavior (221 ± 17, N = 30 switching cycles) and a large number of emitted photons per switching cycle (640 ± 93, N = 30). This is comparable to commonly used SMLM dyes, but in contrast to these compounds, their PFF-1 dye does not need continuous photoactivation to switch states. Also, this nontoxic (HeLa cells) and plasma membrane permeable (mammalian cells) probe could be used in acidic (lysosomes) or neutral pH by tuning the photoactivation step. Optimization can be achieved by adjusting the properties of the laser pulse. For instance, long time-lapse (30 min) SMLM experiments in live HeLa cells could be achieved by repeating the photoactivation pulse (405 nm, 2.6 W cm^−2^, 10 ms) every 10 min to replenish the population of fluxional molecules with an average precision of 33 nm. Even though the acquisition times were long, only modest signs of phototoxicity were observed. Also detailed tracking of single lysosomes, displaying fast directional motion and slow Brownian-like diffusion, was accomplished, which is consistent with previous reports [273]. Combining probe PFF-1 with the mitochondria-targeting triphenylphosphonium group (MitoPFF-1) or the microtubule-targeting group taxol (TaxoPFF-1) resulted in super-resolved images of microtubule and mitochondria, which display neutral or slightly basic pH. Furthermore, 2D and 3D SMLM experiments in neurons showed movement of synaptic vesicles along well-defined tracks and accumulation within hotspots (Figure 22b,c). In addition, the long-time-lapse acquisitions revealed that these vesicles can hop between hotspots using tracks, in which they move more than three orders of magnitude faster than vesicles located at hotspots.

## 3. Synthesis and Structural Aspects

Even though xanthene-based dyes have been known and investigated for about 150 years, their structure-function properties are still not completely understood. Deactivation pathways, solubility and biocompatibility issues, solvent effects, fine tuning of spectroscopic properties and related difficult syntheses are just a few problems. An interesting enhancement of the biocompatibility of rhodamine probes by a neighboring group effect was investigated by Lukinavičius and coworkers [148]. Fluorescent probes are often bound through a linker to a small molecular ligand, that allows targeting the protein of interest. The linker and the fluorophore contribute largely to the final properties of the probe. The result is often a reduced cell permeability or off-targeting [53,163]. Spirocyclic probes are present in their open, zwitterionic and thus more hydrophilic form, or in their spirocyclic hydrophobic form, which shows better cell-permeability [274,275]. Several ways have been investigated to favor the spirocyclic form and hence increase the cell-permeability [167,170,276,277]. The methods include introducing electron-withdrawing groups into the xanthene core or into the benzoic acid substituent. These changes result in a bulkier core structure and alter the physicochemical properties of the dyes. By exploiting the neighboring group effect (NGE), an effect where for example two neighboring carboxy groups can influence each other via steric, electrostatic, or H-bond interactions, the group managed to develop probes with outstanding cell permeability without changing their spectroscopic properties [278]. In contrast to the well-known 5′/6′-carboxyrhodamines the existence of their 4′-isomers was debated for a long time due to synthetic challenges arising from steric hinderance and the altered reactivity of adjacent carbonyl groups related to the ortho-effect [19]. 

**Figure 23:**
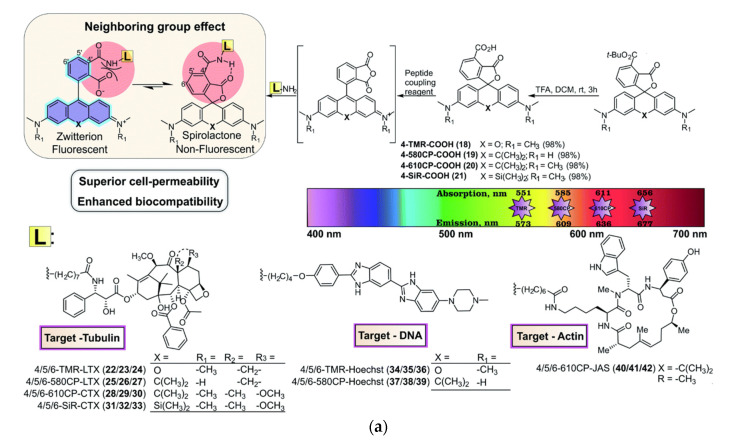

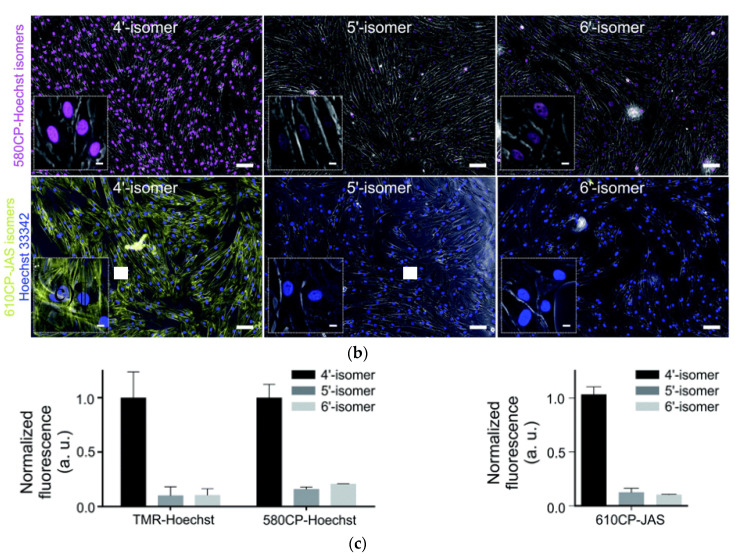
Neighboring group effect related enhancement in biocompatibility: (**a**) **4-TMR-COOH**, **4-580CP-COOH**, **4-610CP-COOH** and **4-SiR-COOH** dyes and conjugates. Their photo-physical properties are depicted in the color spectrum chart and the neighboring group effect is depicted; (**b**) Wide-field microscopy images of living primary fibroblasts, stained with **4/5/6-580CP-Hoechst** (magenta) or **4/5/6-610CP-JAS** (yellow); (**c**) Quantification of DNA probe fluorescence signal in the nuclei. Adapted with permission from [148]—Published by The Royal Society of Chemistry. Scale bars = 100 µm.

The synthetic challenge to produce these 4′-carboxy rhodamines was overcome by reacting a bromo-di-tertbutyl ester with silyl-protected 3,6-dihydroxyxanthones bearing O, C or Si in their 10′-position followed by the late-stage fluorescein to rhodamine conversion strategy to yield TMR-COOH, 580CP-COOH, 610CP-COOH, and SiR-COOH [279]. Interestingly, after the addition of a coupling agent to couple the acid to targeting molecules, the molecule first undergoes intramolecular condensation forming the anhydride (Figure 23a). However, upon addition of an amine, coupling occurs predominantly at the less hindered carboxyl group in the 4′ position. The 4′, 5′ and 6′-regioisomeres show almost identical absorption and fluorescence spectra and quantum yield. The spirolactone and zwitterion state switching ability was determined by measuring the absorbance in water-1,4-dioxane mixtures with known dielectricity constants [280]. After fitting the absorbance change to the dose response equation, the respective ^dye^D_50_ values were obtained. The ^dye^D_50_ value, which indicates the dielectric constant at which the absorbance is reduced to 50%, were similar between most of the regioisomers, only 4-SiR-COOH showed a significantly higher ^dye^D_50_ value. The compounds were bound to either a Larotaxel (LTX) or a Cabazitaxel (CTX) derivative (both anticancer drugs with superior resistance to efflux pumps and blood brain-barrier permeability) to yield tubulin targeting probes TMR-LTX, 580CP-LTX, 610CP-LTX, and SiR-LTX [281,282]. The ^dye^D_50_ values of the 5′- and 6′-regioisomeres were only slightly higher than the corresponding non-tethered fluorophores. However, the ^dye^D_50_ values of the 4′-regiosomeres were significantly increased, implying that the change from carboxyl to amide induces spirolactone formation and thus increase the hydrophobicity and cell permeability of the 4′-probes. The optimized structures of smaller molecular model compounds showed an intramolecular H-bond of 1.75 Å between the carboxamide proton and the carbonyl in the spirolactone in silico. Also, a comparison of total potential energies in 1,4-dioxane and water and NMR experiments confirmed the presence of the NGE. Eventually 4-TNR-LTX, for example, was used to successfully stain tubulin at 1 nM and the centrosome at 1 pM concentration, without a washing step. Furthermore, their probe 4-C610-CTX exceptionally densely labeled microtubule at a resolution of 23 nm in living cells. Hoechst (which targets DNA) and Jasplakinolide (JAS, which targets Actin) were also coupled to their probes and excellent labeling with the 4′-isomers in living cells was achieved (Figure 23b,c).

**Figure 24:**
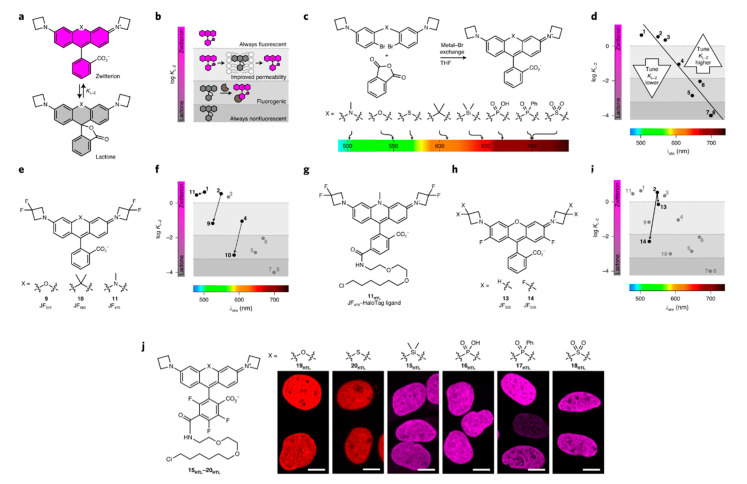
General method to optimize and functionalize red-shifted rhodamine dyes: (**a**) the lactone–zwitterion equilibrium of JF rhodamine dyes and the equilibrium constant K_L-Z_; (**b**) Phenomenological plot categorizing the properties of different dyes based on K_L-Z_; (**c**) Synthesis, structures and λ_exc_ of dyes 1–8; (**d**) Plot of K_L-Z_ versus λ_exc_ for dyes 1–8 and general tuning strategies for dyes with short or long λ_exc_; (**e**) Structures of dyes 9–11; (**f**), Plot of K_L-Z_ versus λ_exc_ showing decreased K_L-Z_ for dyes 9–11; (**g**) Structure of JF_479_–Halotag ligand (11_HTL_); (**h**), Structures of dyes 13–14; (**i**) Plot of K_L-Z_ versus λ_exc_ showing decreased K_L-Z_ for dyes 13–14; (**j**) High-magnification images of fixed U2OS cell nuclei expressing Halotag–histone H2B and labeled with **15_HTL_**–**20_HT_**_L_. Adapted by permission from Nature: Springer, Nature Methods [51] Copyright (2020). Scale bars = 10 µm.

A general method to optimize and functionalize red-shifted rhodamine dyes was established by the group of Lavis and coworkers [51]. Recently the group already optimized a general method to improve and fine-tune rhodamine fluorophores by incorporating four-membered azitidines into the structure and called them Jenelia Fluor (JF) dyes [52]. This method allowed them to optimize blue- to red-excited rhodamines but could not be applied to near-infrared or far-red analogues due to their tendency to adopt a colorless form. Their new approach makes use of their previously established K_L-Z_ (lactone-zwitterion equilibrium constant) which can be used to estimate the molecules performance in biological systems (Figure 24a) [52,277,283,284]. Dyes with high K_L-Z_ predominantly exist in their zwitterionic form, while rhodamines with modest K_L-Z_ values show better cell penetration ability since a higher ratio of the dye takes the spirolactone form and dyes with low K_L-Z_ values are often used as fluorescent dyes, as binding to their targets often shifts the equilibrium to their fluorescent open form (Figure 24b). Some dyes with extremely low K_L-Z_ values only exist in their spirolactone form and are thus unusable in biological experiments. Their group synthesized different rhodamine systems with carbon, oxygen, silicon, sulfur, sulfone (X = SO_2_), phosphinate (X = PO_2_H), phosphine oxide (X = P(O)Ph) and nitrogen (X = NCH_3_) in position 10′, measured their absorption maximum (λ_exc_), fluorescence emission maximum (λ_em_), fluorescence quantum yield (Φ) and their extinction coefficients (ε) in aqueous buffer and their K_L-Z_ values in a dioxane-water mixture (Figure 24c). They discovered an inverse correlation of K_L-Z_ and λ_exc_, probably due to the electron-withdrawing character of the 10′-atoms which was also demonstrated by Liu and coworkers for O, C(CH_3_)_2_ and Si(CH_3_)_2_ and will be discussed in the next section (Figure 24d) [285,286]. Lowering the K_L-Z_ value would improve tissue permeability and render them usable as fluorescent probes as mentioned above. One way to do that is incorporating 3,3-difluoroazetidies, which also results in a hypsochromic shift of about ≈25 nm, transforming for example JF_459_ (K_L-Z_ = 3.5) into JF_525_ (K_L-Z_ = 0.68) (Figure 24e,f). Their probe 11_HTL_ based on JF_525_ and HaloTag showed improved cell permeability and is blood-brain-barrier permeant (Figure 24g). Another way to decrease K_L-Z_ involves direct fluorination on the xanthene system and is complementary with the incorporation of 3,3-difluoroazetidines (Figure 24h,i). On the other hand, the group also developed a method to improve the far-red and NIR rhodamines in regard of their low K_L-Z_ values. They had previously shown that halogenation on the pendant phenyl ring system can increase the K_L-Z_ value by lowering the pK_a_ value of the benzoic acid moiety [287]. This strategy was demonstrated on all four far-red dyes and resulted in dyes with significantly higher K_L-Z_ values. In this context, the group furthermore discovered, that nucleophiles like N_3_-, CN-, NH_3_ or NH_2_OH readily add to the fluorinated ring with a regioselectivity at the 6 position. Even though it has been known and utilized for a long time that thiols take part in that reaction, the reactivity with other nucleophiles was largely unexplored. Novel 6-carboxy-4,5,7-trifluororhioamines were synthesized and coupled to HaloTag (**15_HTL_**–**20_HTL_**) and SNAPtag and resulted in excellent live-cell labels with low nonspecific staining (Figure 24j). 

Time-resolved spectroscopy is of great interest, as it allows us to monitor interactions between biomolecules in real-time. The necessary continuous excitation of these fluorophores, however, show deleterious processes of photobluing or -redding (hypso- or bathochromic shifts of their excitation and emission maxima), blinking (temporal conformation change to a non-emissive state), and photobleaching (irreversible decay of the fluorophore) [288,289]. The presence of naturally abundant oxidizing (dissolved oxygen) or reducing species (glutathione) in cells promote these processes [290]. While the resulting shifted absorbance and fluorescence maxima after photobluing or -redding can lead to a misinterpretation of the collected data, especially during multi color nanoscopy, where blinking and photobleaching lead to a loss of signal intensity. Hell and coworkers investigated methods to reduce these shifts in the case of xanthone based probes [291]. 

**Figure 25:**
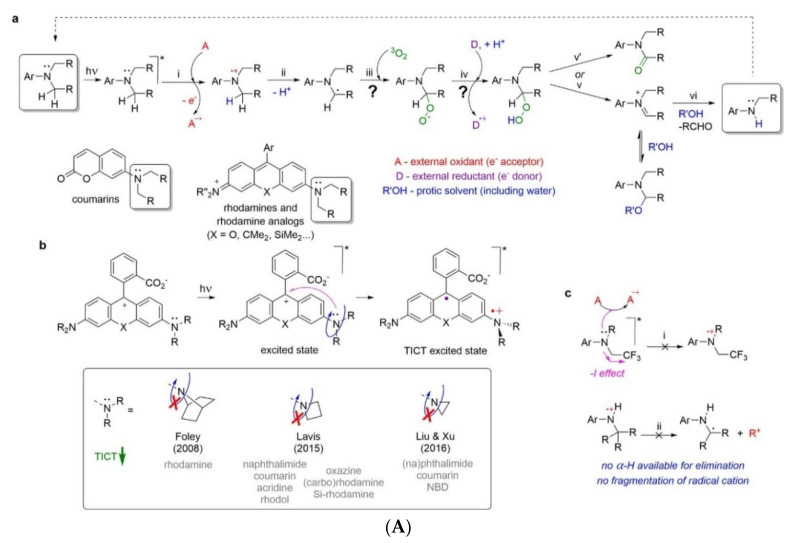

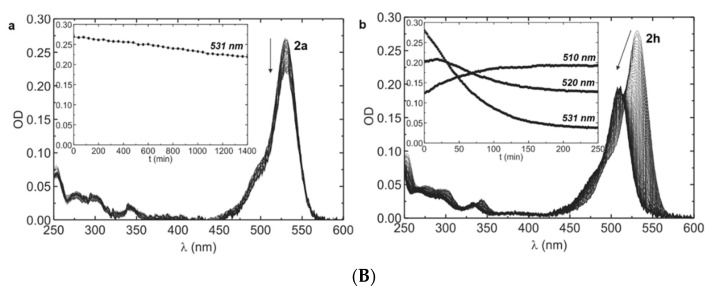
Strategies to reduce photobluing: (**A**) (**a**) General oxidative photobluing pathway for fluorescent dyes with (di)alkylamino auxochromic groups; (**b**) Strategies aimed at suppression of transitioning into the twisted intramolecular charge transfer (TICT) excited state for select classes of fluorophores; (**c**) Possible approaches to mitigate the dye photobluing unrelated to TICT suppression; (**B**) Stepwise Time-resolved absorption spectroscopy experiment demonstrating photolysis of (**a**) a photooxidation-resistant, nonbluing fluorophore in comparison with (**b**) a photooxidation-prone fluorophore. Adapted with permission from [291]. Further permissions related to the material excerpted should be directed to the ACS.

The stepwise N-dealkylation of rhodamines leads to a hypsochromic shift of their absorption and emission maxima (10–15 nm per dealkylation step). The mechanism proceeds through the initial formation of a radical cation, which will then be deprotonated to form an α-aminoalkyl radical (Figure 25(Aa)). This species loses an alkyl substituent in the form of an aldehyde in oxygen containing media, via a hemiaminal, peroxyaminal or an iminium type species. Related to this oxidative photodealkylation process is the transition into a twisted intramolecular charge transfer (TICT) excited state, which corresponds to an internal electron transfer from the alkylamino residue to the xanthylium core, first described by Foley and improved by Lavis, Lin and Xu (Figure 25(Ab)) [284,292,293]. By introducing bridged 7-azabicyclo[2.2.1.]heptane, azetidine or aziridine substituents this process can be reduced. Other than TICT suppression, changing the alkyl group of the amine to a moiety that hinders the first one-electron photooxidation step, or the second α-deprotonation of the radical cation step should result in more photostable dyes (Figure 25(Ac)). The Hell group successfully synthesized a range of different N,N′-di-tert-alkylrhodamines by an Ullmann-type amination of 3′,6′-dihalofluorans. Their photophysical properties were compared to reference rhodamines (i.e., TMR, JF_525_, JF_549_). The synthesized N,N′-di-tert-dialkylrhodamines were generally bright fluorophores (ε = 10^5^ M^−1^cm^−1^, Φ > 90%) with excited state lifetimes of up to 4 ns. The photooxidative degradation of these dyes was tested in air-saturated acidified ethanol (with 0.1 *v*/*v*% TFA) under continuous irradiation. Furthermore, dealkylation products were identified by (HR-)MS, NMR and UV-vis spectroscopy. For instance, all five dealkylation products from Rhodamine B to Rhodamine 110 (resulting in a total hypsochromic shift of ≈50 nm) could be separated and identified, one of them being N-acetylrhodamine 110. In general, dyes bearing a tert-alkyl substituent did not show detectable photobluing effects (Figure 25B). Rhodamine 110 showed a similar spectral stability, also azabycycloheptane-substituted dyes showed resistance. Dyes like JF_525_ and JF_549_ underwent degradation resulting in aldehyde bearing derivatives. This could be problematic for long-term microscopy in living cells since unwanted cross-reactivity with biomolecules can be observed.

An interesting synthetic approach for a diversity-oriented library for wide spectrum bactericidal rhodamines has been developed by Yang and coworkers [294].

**Figure 26:**
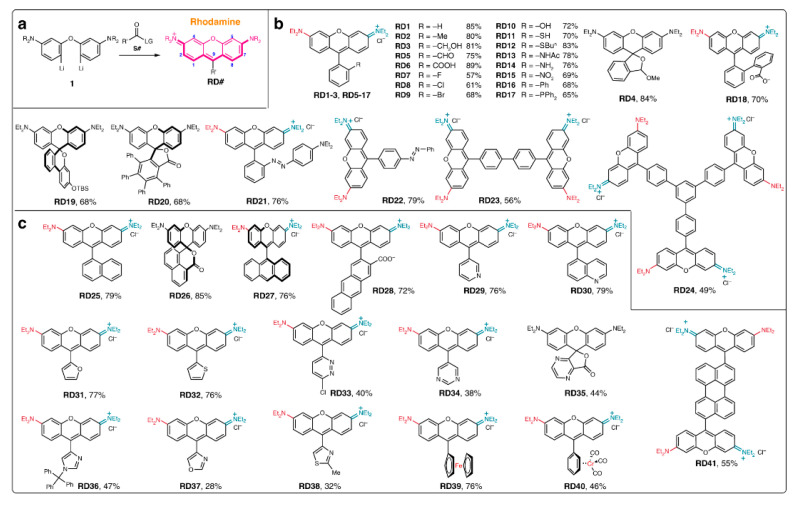
An excerpt of the synthesized rhodamine library via nucleophilic condensation of a dilithium reagent with various substrates: (**a**) The general synthetic pathway; (**b**) Structures of rhodamine dyes with phenyl groups at C-9 (RD1-RD24); (**c**) Structures of rhodamine dyes with polyaromatic or heteroaryl groups at C-9 (RD25-RD41). Adapted with permission from [294].

Even though their best hits were not spirocyclic rhodamines their synthesis affords a wide range of easily accessible spirocyclic rhodamines that might be useful in other applications (Figure 26). The developed strategy consists of a nucleophilic condensation of a dilithium reagent with readily available esters, anhydrides or even amides. They were able to synthesize dyes with exotic substituents bound to the C-9 carbon as phenyls, aryls, alkyls, alkenyls, and acyls. Shortly after, the group of Lavis and the group of Sparr also reported this synthetic approach [287,295]. The yields were generally higher than 50% and the substrate scope can possibly be expanded to other functional groups as anhydrides, lactones, or lactams.

**Figure 27:**
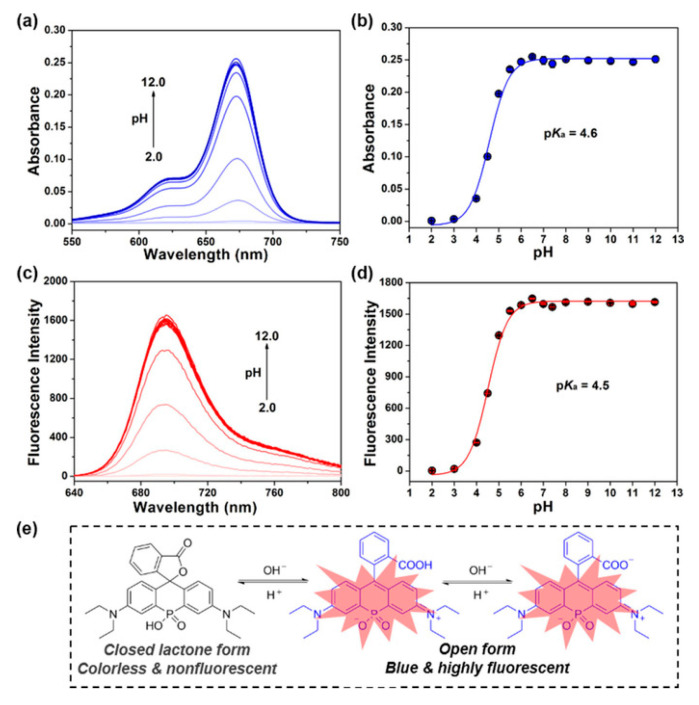
The pH-dependent photophysical properties of the new dye; (**a**) Absorption and (**c**) fluorescence with pH and the resulting pK_a_ titration curves (**b**,**d**); (**e**) Proposed closed–open equilibrium of the spirolactone. Adapted with permission from [296].

A multicolor palette is desired, since then a multi probe approach can be utilized, and different targets can be visualized simultaneously. It is crucial to expand the color palette towards the red and IR part of the spectrum which also increases the penetration depths of the skin. The Wang group tried to broaden the properties of phosphorous substituted rhodamines which emit in the NIR region [296]. Most xanthene-based dyes use their aniline- or phenol-moiety or their spiro-ring to manipulate their fluorescence after reaction with an analyte has occurred. So far not much attention had been paid to caging the bridging atom, so the group developed a novel scaffold of organophosphorus rhodamines with NIR emission wavelengths (λ_em_ = 690–755 nm). Interestingly, these bright, blue-colored fluorescent dyes turn colorless at acidic pH (<5.5) (Figure 27). This reactivity probably originates from the electron-withdrawing capacity of the phosphorous group, that locks the molecule in its spirocyclic form, which is also supported by quantum mechanical calculations. Furthermore, the diarylphosphoric acid moiety can be coupled to a caging group which renders the molecule colorless and upon reaction with an analyte the fluorophore exhibits strong fluorescence. This was shown by coupling the fluorophore to three different caging molecules: 4,5-dimethoxy-2-nitrobenzyl (DMNB, photocleavable at λ = 365 nm), 4-benzylboronic acid pinacol ester (reacts with H_2_O_2_) and 4-nitrobenzyl (targets nitroreductase, which can be overexpressed in tumors because of hypoxia). In all cases, the caging groups were removed by the targets in vivo and strong fluorescence of the dyes with only low cytotoxicity was observed.

Shortly after Wang et al., Stains and coworkers reported similar P-bridged molecules. Even though they do not address spirocyclic compounds the synthesis should be applicable. Previously, the group had already replaced the bridging atom of a xanthene dye with phosphorous to synthesize a dye with a phosphinate functionality [297]. The dye called Nebraska Red (NR) retained brightness and photostability of the rhodamine scaffold, while displaying >110 nm shifts in excitation and emission (Table 1).

**Figure 28:**
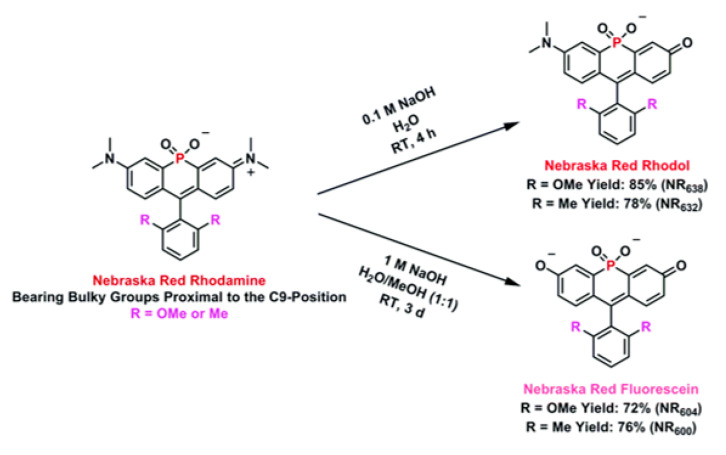
Direct transformation of NR rhodamine to NR rhodol or NR fluorescein. Adapetd with permission from [297].

They further increased the chemical functionality of NR dyes through the synthesis of other fluorescein-based derivatives, namely rhodamine, rhodol and fluorescein [299]. Interestingly the synthesis includes the reaction of the Rhodamine with NaOH to yield the rhodol and the fluorescein respectively under mild basic conditions (Figure 28). This is a stark contrast to the behavior of regular O-bridged rhodamines for example, where the reaction requires harsher conditions, such as refluxing in 2 M KOH for several days with only about 30% yield. Moreover, the formation of the fluorescein product is not observed. The resulting phosphinates showed excellent spectroscopic behavior and no evidence of photobleaching even after continuous irradiation. Similar to the aforementioned article the group used the phosphinate to bind a substrate. As a substrate they chose acetoxymethyl (AM) which can readily be cleaved by cellular esterases. Their probe diAM-NR_600_ showed an up to 10-fold increase in fluorescence in in vivo. This fluorescein like dye showed the highest brightness but its pK_a_-value was measured to be 6.75. The group is now trying to lower its pK_a_ to further increase its brightness since a large ratio is non-fluorescent at physiological pH.

## 4. Computational Approaches

Even though there are plenty of probes available and guidelines on how to fine tune and rationally design the pK_cycl_ value of spirocyclic probes, it is still mostly a trial-and-error approach to end up with a molecule with the desired properties. Synthesis is often time consuming and involves many steps to introduce a certain group into a molecule which often must be already present in the starting materials. Computational chemistry has become a crucial part of today’s research and it can help to predict equilibrium constants of spirocyclization of novel molecules as our group has shown recently for hydroxymethyl rhodamines (HMR) [44].

**Figure 29:**
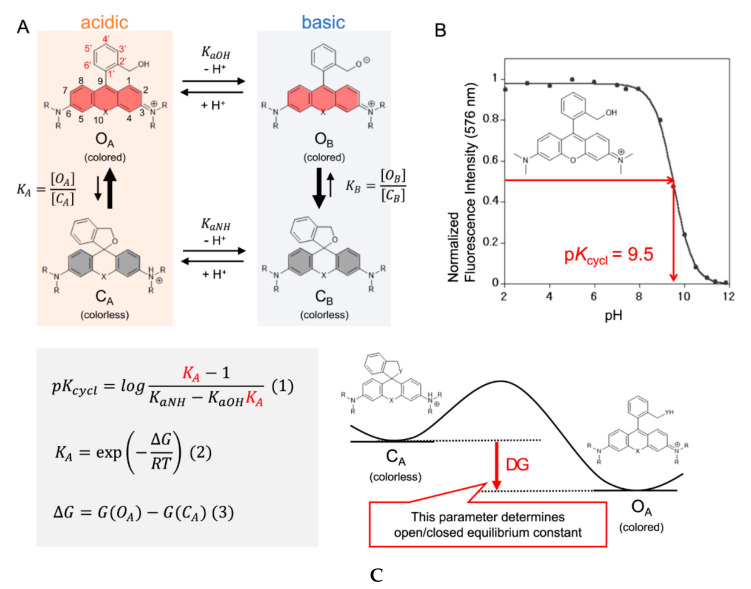
Intramolecular spirocyclization of HMR derivatives. (**A**) Acid–base equilibrium of HMR derivatives; (**B**) Correlation between pH and normalized fluorescence intensity of HMTMR; (**C**) Formula for pK_cycl_ based on statistical mechanics and the visualization of ΔG. Adapted with permission from [44].

This might help to revolutionize design strategies and hence eliminate time-consuming synthesis of multiple candidates. The equilibrium of the open and the closed form is pH-dependent and the pK_cycl_ values of known HMR derivatives were used to quantum chemically predict novel probes. Assuming that only four species (open forms under acidic O_A_ and basic conditions O_B_ and closed forms under acidic C_A_ and basic C_B_ conditions) were involved in this equilibrium the pK_cycl_ value can be interpreted as the pH value at which the concentration of the open forms (O_A_ + O_B_) is equal to that of the closed forms (C_A_ + C_B_) (Figure 29A,B). This leads to the equation in Figure 29C which can be used to predict pK_cycl_ values by quantum chemically estimating the difference in free energy ΔG between the open form and the closed form. The equilibrium constants K_aOH_ and K_aNH_ can be replaced by reported pK_a_ values of similar structures (benzyl alcohol, K_aOH_ = 10^−15.4^; aniline, K_aNH_ = 10^−4.6^; N,N-dimethylaniline, K_aNH_ = 10^−4.9^).The calculations were performed at the B3LYP/6-31G(d) level with water included in the PCM model. We found that it is crucial for accurate calculations to introduce first shell water molecules in the calculations, and that a three-water bridge between the nucleophilic hydroxymethyl group and the amino groups of the xanthene ring gave results in very good agreement with previously reported values of known rhodamine probes. These results are interesting, since the proton moves from the nucleophile to the amino group during the spirocyclization reaction. The activation free energy of this reaction of HMTMR was thus estimated to be 28.7 kJ mol^−1^ by IRC calculations which suggests the reaction can proceed spontaneously at room temperature [300]. Next, we calculated pK_cycl_ values of molecules bearing F, Me, CF_3_ or H moieties in positions 3′, 4′, 5′ and 6′ of the benzyl ring, that have never been synthesized before (Table 2). After synthesizing a few of these structures, their measured pK_cycl_ values were in good agreement with their predicted values. The trend of the introduced groups suggests a strong impact on lowering the pK_cycl_ value by substituents on position 3, regardless of their electron-donating- or withdrawing effects. The bulkiness of the moieties next to the attacking nucleophile seems to play a bigger role.

The developed prediction method was then used to design and synthesize two novel red-shifted HMR derivatives (HMRR with Si and HMRY with C in position 10′) with appropriate pK_cycl_ values for their use as GGT probes. The measured pK_cycl_ values fit the predicted values very well and their γ-glutamate-bound analogues (gGlu-HMRR and gGlu-HMRY) were successfully used as GGT-probes to visualize tiny tumors in a mouse model in vivo.

It is not only difficult to predict the pK_cycl_ values of novel fluorophores but also their blinking properties, which is an important parameter for SML microscopy, one of the most frequent methods for super-resolution imaging [301].

**Figure 30:**
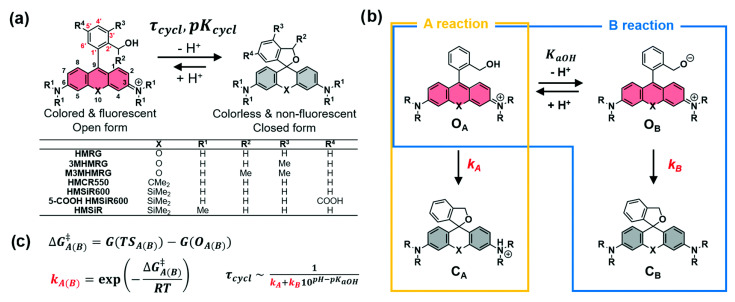
Computational approach to predict blinking properties of spirocyclic dyes: (**a**) Thermal equilibrium of HMR derivatives based on intramolecular spirocyclization between the fluorescent open form and the non-fluorescent closed form. τ_cycl_ is the lifetime of the open form. pK_cycl_ is the equilibrium constant of intramolecular spirocyclization; (**b**) Model mechanism of ring-closing reaction of HMR derivatives; (**c**) Formula for τ_cycl_ based on statistical mechanics and transition-state theory. Adapted with permission from [45].

Our group recently also developed a quantum-chemical calculation-based method to predict the equilibrium constant and blinking kinetics of the intramolecular spirocyclization of novel compounds [45]. To develop spontaneously blinking, spirocyclic fluorophores the pK_cycl_ value should be below 6, so that only a small subset of fluorophores exists in the open state at the physiological pH of 7.4 and the lifetime of the open form (τ_cycl_) should be in the range of one to hundreds of milliseconds, to match the exposure time of the used microscopes [42]. The pK_cycl_ values were determined by the beforementioned quantum-chemical method [44]. We assumed that the second important parameter τ_cycl_ can be predicted by calculating the activation free energy (ΔG^‡^) of the intramolecular spirocyclization reaction. To calculate the activation free energy of the reaction we previously showed that the τ_cycl_ value of HMSiR changes depending on pH. We proposed two processes of the ring-closing reaction, a pH-independent process (A reaction, which yields k_A_) and a pH-dependent process (B reaction which yields k_B_), that could be used to calculate τ_cycl_ via the mentioned formula (Figure 30a–c). The calculated values were in good agreement with experimentally measured values. Next, we identified three promising candidate scaffolds for spontaneously blinking fluorophores M3MHMRG, HMCR550, and HMSiR600. The spectroscopic properties of these fluorophores matched well with our calculations (Table 3).

Live-cell imaging was also performed by applying 4′- and 5′-Halo-HMCR550 to Vero cells expressing β-tubulin-HaloTag. Even though tubulins were specifically labeled with 5′-Halo-HMCR550, super resolution reconstruction showed cluster-like inhomogeneous structures, probably due to a too high emitter density. We evaluated the pK_cycl_ value of HaloTag-5′-Halo-HMCR550 and found it to be 7.0, 1.8 units higher than that of non-conjugated 5′-Halo-HMCR550, which is explainable through dye-protein interactions. The regioisomer 4′-Halo-HMCR550 on the other hand showed good cell permeability, and super-resolution reconstructed images of microtubule could be obtained.

**Figure 31:**
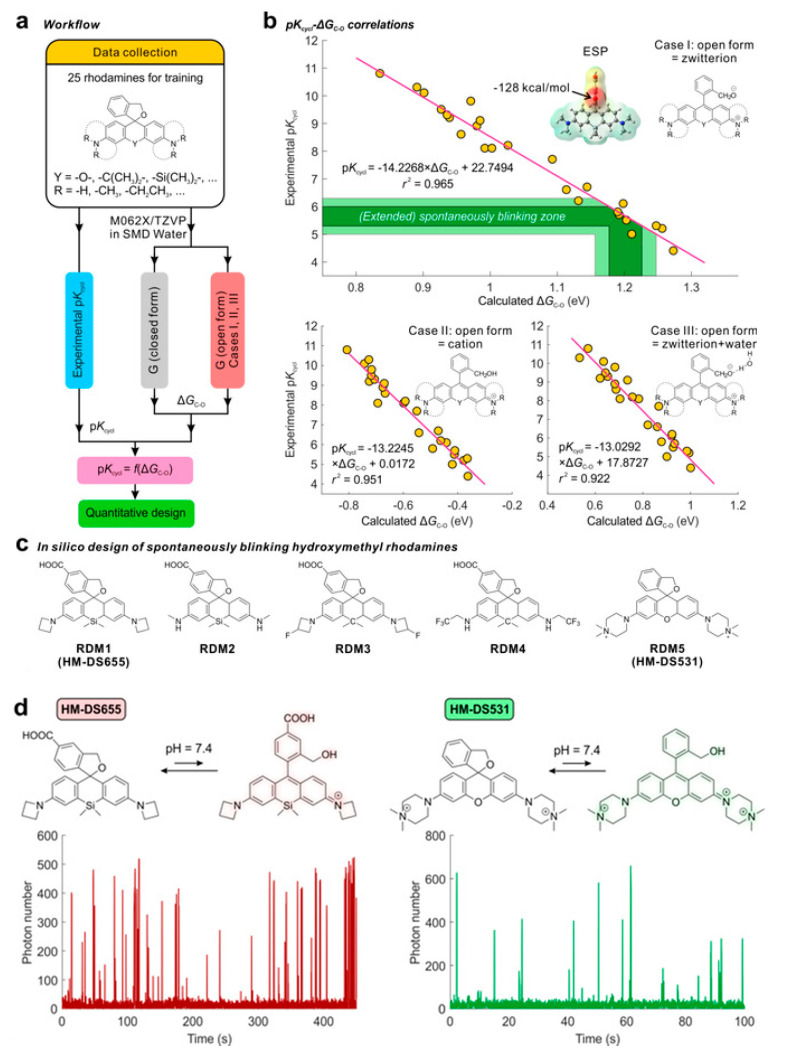
Computational approach to predict the pK_cycl_ value of blinking dyes: (**a**) The workflow in this work; (**b**) Correlations between calculated ΔG_C-O_ and experimental pK_cycl_ values for a series of hydroxymethyl rhodamine dyes reported by our group; three types of open forms were considered: zwitterion, cation, and zwitterion + water; (**c**) Molecular structures of computationally designed spontaneously blinking rhodamines RDM1-RDM5 with synthesized candidates **HM-DS655** and **HM-DS531**; (**d**) blinking of **HM-DS655** and **HM-DS531.** Adapted with permission from [302].

Another approach to facilitate rational design of spontaneously blinking rhodamine probes was developed by Liu and coworkers [302]. In their approach they used the ΔG_C-O_ value, which is the Gibbs free energy difference between the open and the closed forms similar to our previously discussed approaches. The group hypothesized that there is a correlation between calculated ΔG_C-O_ and experimental pK_cycl_ values. To verify their assumption, they collected the pK_cycl_ values of 25 existing rhodamine dyes reported by our group and calculated their ΔG_C-O_ values at M06-2X/TZVP level in water [42,116,121,303,304,305]. In contrast to our model, they used zwitterionic molecules in their calculations and it did not necessitate the inclusion of explicit water bridges or water molecules (Figure 31a,b). Interestingly, a well-fitting correlation was found with only 0.3 pH values of mean error. Due to that calculation error the spontaneously blinking window was set from 5.3–6.0 to a permissible pK_cycl_ range from 5.0–6.3 which translates to ΔG_C-O_ values of 1.156–1.284 eV. With this in hand, the group designed five potential spontaneously blinking rhodamines of different colors and synthesized two of these (Figure 31c). Both dyes HM-DS655 and HM-DS531 showed experimental pK_cycl_ values (5.3 and 5.3 respectively) well in agreement with the predicted values (5.65 and 5.05 respectively).

SMLM experiments demonstrated that these molecules indeed showed blinking behavior with lifetimes of 90 ms for HM-DS655 and 50 ms for MH-DS531 in PMMA films (Figure 31d). Laser flash photolysis experiments in PBS buffer revealed lifetimes of 1.47 ms for HM-DS531 and 100 ms for HM-DS655 which is compatible with the imaging acquisition speeds of existing detectors. Eventually, HM-DS655 was bound to SNAP to form HM-DS655-SNAP which was used to stain mitochondria in fixed HeLa cells, transfected with pSNAPf-Cox8A. The dye demonstrated excellent spontaneous blinking properties in 3D-STORM experiments and good photostability up to a sample depth of about 600 nm. An average photon number of 1216 of a single molecule per frame was determined which results in an average optical resolution of 19.3 nm. Interestingly, the group also found a linear correlation between ΔG_C-O_ values and logK_L-Z_ values. Even though they were able to predict potential candidates by correlating the ΔG_C-O_ value to its pK_cycl_ value, their approach does not allow for prediction of the estimated blinking frequencies (τ_cycl_).

**Figure 32:**
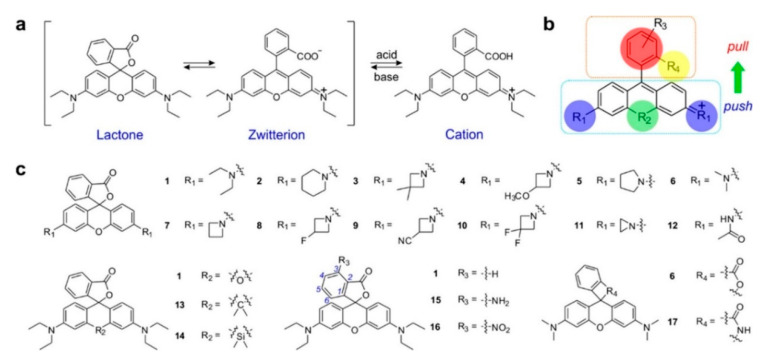
Development of a unified push-pull model: (**a**) Lactone−zwitterion−cation equilibrium of rhodamine B; (**b**) Four representative substitution sites of rhodamine derivatives that affect lactone-zwitterion-cation equilibrium; (**c**) Molecular structures of representative rhodamine dyes 1−17 used for the calculations. Adapted with permission from [286]. Copyright (2020) American Chemical Society.

Another interesting quantum chemical contribution of Liu and coworkers in collaboration with the Xu group was the development of a unified push-pull model for understanding of the ring-opening mechanism of rhodamine dyes [286]. It is known that highly polar solvent and low pH facilitates ring-opening, and that solvents with hydrogen-bond acceptor groups play a significant role, which is also confirmed by DFT calculations [33,38,306,307,308]. Nowadays, many dyes have been synthesized and it has been empirically shown that increasing the electron donating strength of the amino group is enhancing ring-opening, while exchange of the bridging oxygen to Si or C reduces the tendency for ring opening [42,50,51,52,309,310]. Furthermore, electron-withdrawing groups in the meso-phenyl ring position promote ring-opening whereas a change of the internal nucleophile can hinder the formation of the zwitterionic form [42,311,312]. To establish a unified mechanistic model, to help to rationally design rhodamine dyes, the group carried out DFT calculations with M062X functional and TZVP basis set on ≈200 conformations and configurations of 24 representative rhodamine dyes. The ring-opening reaction was found to take place between the lactone and the zwitterion of rhodamine B. Essentially, the ring-opening reaction is accompanied by a considerable amount of change in the molecule’s polarity. The relatively non-polar lactone (calculated overall dipole moment 10.35 Debye) changes into a highly polar state (21.91 Debye). Since the ring-opening reaction is a thermodynamically driven process, stabilization of the highly polar zwitterion reduces its relative free energy. To stabilize that polar tautomer, a push-pull effect is needed, which can be tuned either by structural modification of the molecule (intrinsic factors) or by adjusting the experimental conditions (environmental factors). The intrinsic factors are mainly influenced by four types of substituents in the molecule R_1_–R_4_ (Figure 32). R_1_ and R_2_ are modifications on the xanthene moiety, which can donate charge to the phenyl-ring and serve as an electron donor in the push pull model. Increasing the electron donating ability of R_1_ enhances the push-pull effect, thus stabilizing the zwitterion and hence leading to a higher pK_a_ of the molecule. This was shown by calculations resulting in a reduced energy barrier for the lactone to open its ring. The effect of the R_2_ group also depends on its electron-donating strength and hence the electronegativity of the group. The electronegativities of O (3.44), C (2.55), and Si (1.90) are consistent with a lowering of the respective pK_a_ values in experimental observations. On the other hand, raising the electron-withdrawing strength of R_3_ enhances the push-pull effect and leads to a lower energy barrier. The spiro unit R_4_ also greatly impacts the equilibrium, the higher the electron-withdrawing effect of R_4_ the stronger the push-pull effect. To summarize this part, R_1_ and R_2_ are part of the push effect and R_3_ and R_4_ are part of the pull effect.

Apart from those intrinsic factors the environmental factors also greatly influence the equilibrium. High polarity solvents enhance the push-pull effect and stabilize charge separated zwitterions through dipole-dipole and induced-dipole interactions. Calculations support experimental observations that a solvents polarity is not as important as its hydrogen bond donating strength. Although DMSO is a polar solvent, dissolved zwitterions have a significantly higher energy than lactones; hence in DMSO dissolved rhodamine B remains colorless. Not surprisingly, the pH value also effects the open-close equilibrium by stabilizing the negative charge in acidic medium via protonation. In a similar way, complexation with metal ions (Lewis acids) like Pb^2+^ or Cu^2+^ stabilizes the zwitterionic state, which has been used to detect metals in solution.

## 5. Conclusions and Perspective

In this review, we summarize the research progress on small, spirocyclic xanthene-based fluorescent probes within the last three years. For 150 years spirocyclic xanthene dyes have become an essential tool for modern research. This review covered only a fraction of possible applications and recent publications in the field. Even though these probes have been used for a long time, many uncertainties remain. Researchers like Lavis, Lukinavičius, Johnsson, and Hell have helped to understand and cope with effects such as photobluing, cell permeability, and how to fine tune probes to hold the best possible properties. Computational chemistry is by far no new technology, but it only recently became of great interest for predicting suitable candidates without the need for tedious syntheses, as shown by us and the Liu group. This might lead to novel probes that will perform their task more efficiently, resulting in brighter, more precisely located, less toxic, and at higher wavelengths absorbing fluorophores. With nitrogen and phosphate, almost all possible bridging atoms have already been introduced, but this may not be the end of their optimization as different oxidation states or bound groups might also have a great influence. A combination of these new findings may facilitate the development of probes that can easily be used in vivo, which still remains an ongoing challenge.

## Figures and Tables

**Table 1:** Summary of spectroscopic data of the newly synthesized dyes, including previously reported values for Tokyo Magenta [298] and Tokyo Green [98] for comparison.

Dyes		*λ*_abs_ [nm]	*λ*_em_ [nm]	Stokes Shift [nm]	ε [M^−1^cm^−1^]	Φ	Brightness (ε∙Φ) [M^−1^cm^−1^]	pK_a_
Rhodamine	NR_675_	675	695	20	73,200	0.24	17,568	<0.52
	NR_669_	669	689	20	77,200	0.30	23,160	<0.59
Rhodol	NR_638_	638	662	24	55,630	0.21	11,682	5.16
	NR_632_	632	655	23	63,970	0.26	16,632	5.20
Fluorscein	NR_604_	604	627	23	45,740	0.48	21.955	6.71
	NR_600_	600	619	19	67,570	0.68	45,948	6.75
	Tokyo Magenta	582	598	16	110,000	0.42	46,200	6.8
	Tokyo Green	491	510	19	85,000	0.85	72,250	6.2

**Table 2:**
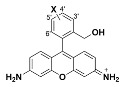
Calculated and measured pK_cycl_ values of HMRG derivatives substituted with various groups on the benzene moiety. R = H; X = O.

	Exp. p*K*_cycl_	Calc. p*K*_cycl_			
X	3′ (5′)	3′	4′	5′	6′
F	8.0	8.1	8.3	7.4	8.1
Me	6.6 (8.2)	6.1	8.1	7.9	7.2
CF_3_	5.3	5.3	7.1	7.4	7.6
H	8.1	7.9	-	-	-

**Table 3:** Photophysical properties of spontaneously blinking fluorophores with calculated and experimental values.

	*λ*_abs_ [nm]	*λ*_em_ [nm]	Exp. p*K*_cycl_	Calc. p*K*_cycl_	Exp. *τ*_cycl_ [ms]	Calc. *τ*_cycl_ [ms]
HMRG	500	523	8.1	7.9		-
3MHMRG	500	523	6.6	6.1	27.3	38.0
M3MHMRG	498	520	4.7	<4	6.6	2.0
HMCR550	559	582	5.4	5.7	40.9	78.6
HMSiR600	595	615	4.4	<4	-	5.3
5-COOH HMSiR600	595	615	4.7	-	11.5	-
HMSiR	650	671	5.8	6.2	375	165
